# Supplementary material for: VeVaPy, a Python Platform for Efficient Verification and Validation of Systems Biology Models with Demonstrations Using Hypothalamic-Pituitary-Adrenal Axis Models
Source: Entropy (Basel). 2022 Nov 29;24(12):1747. doi: 10.3390/e24121747 (PMC9777964; doi:10.3390/e24121747)
Supplement: Supplementary file 1 [file entropy-24-01747-s001.zip › Supplementary Files/Manuscript_Supplementary_Figures.pptx]

## Slide 1
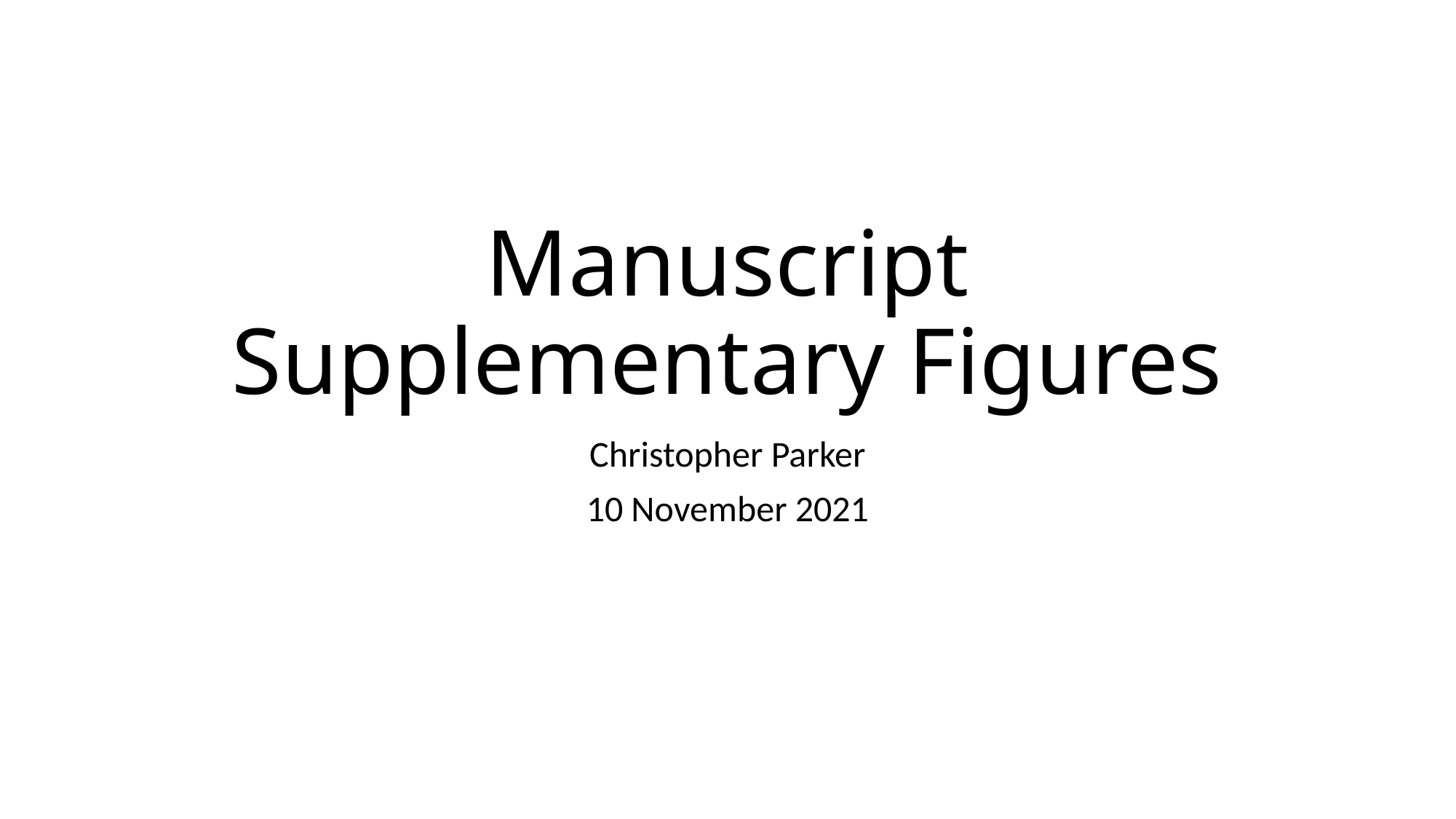

# Manuscript Supplementary Figures
Christopher Parker
10 November 2021

## Slide 2
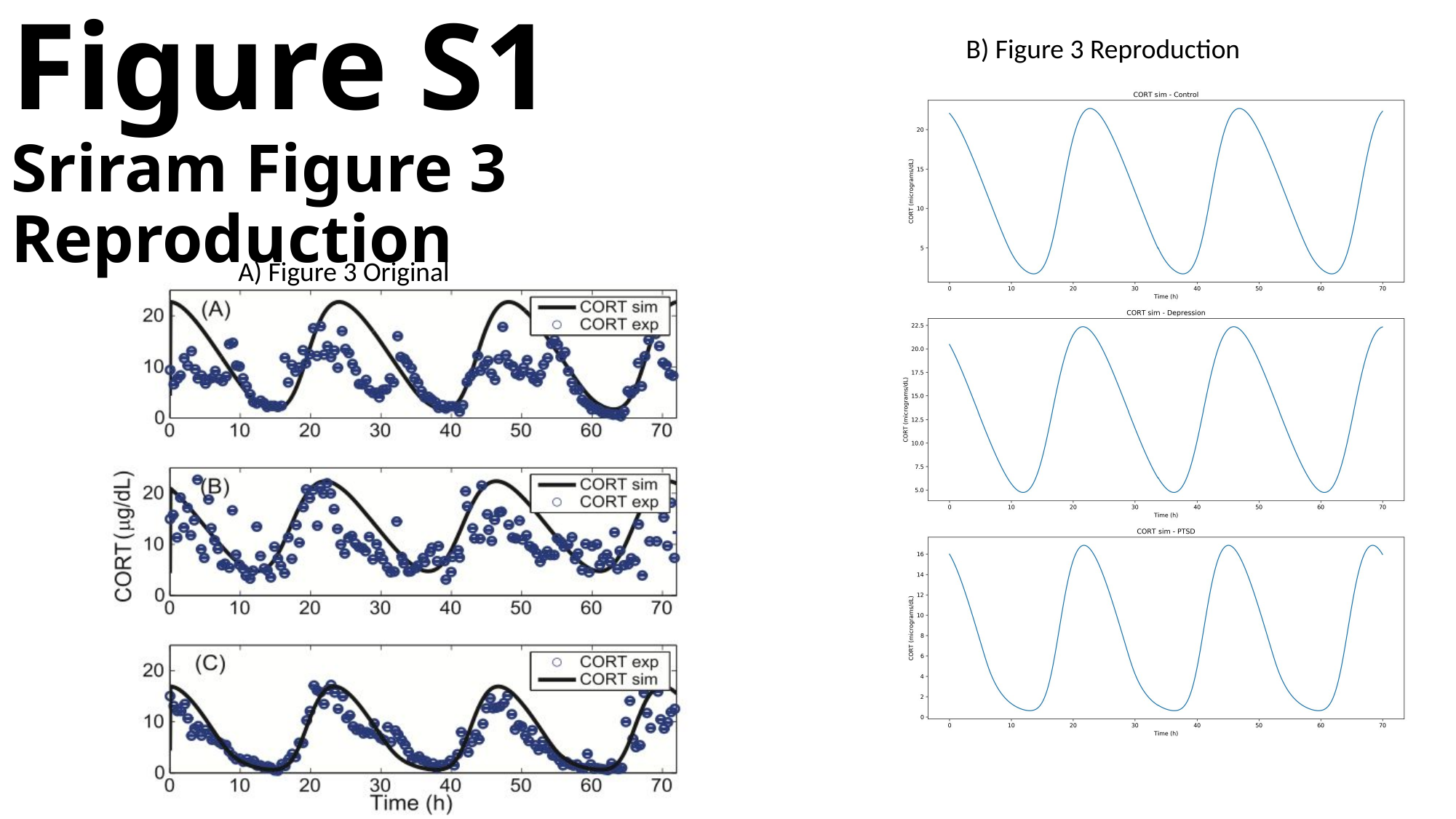

# Figure S1 Sriram Figure 3 Reproduction
B) Figure 3 Reproduction
A) Figure 3 Original

## Slide 3
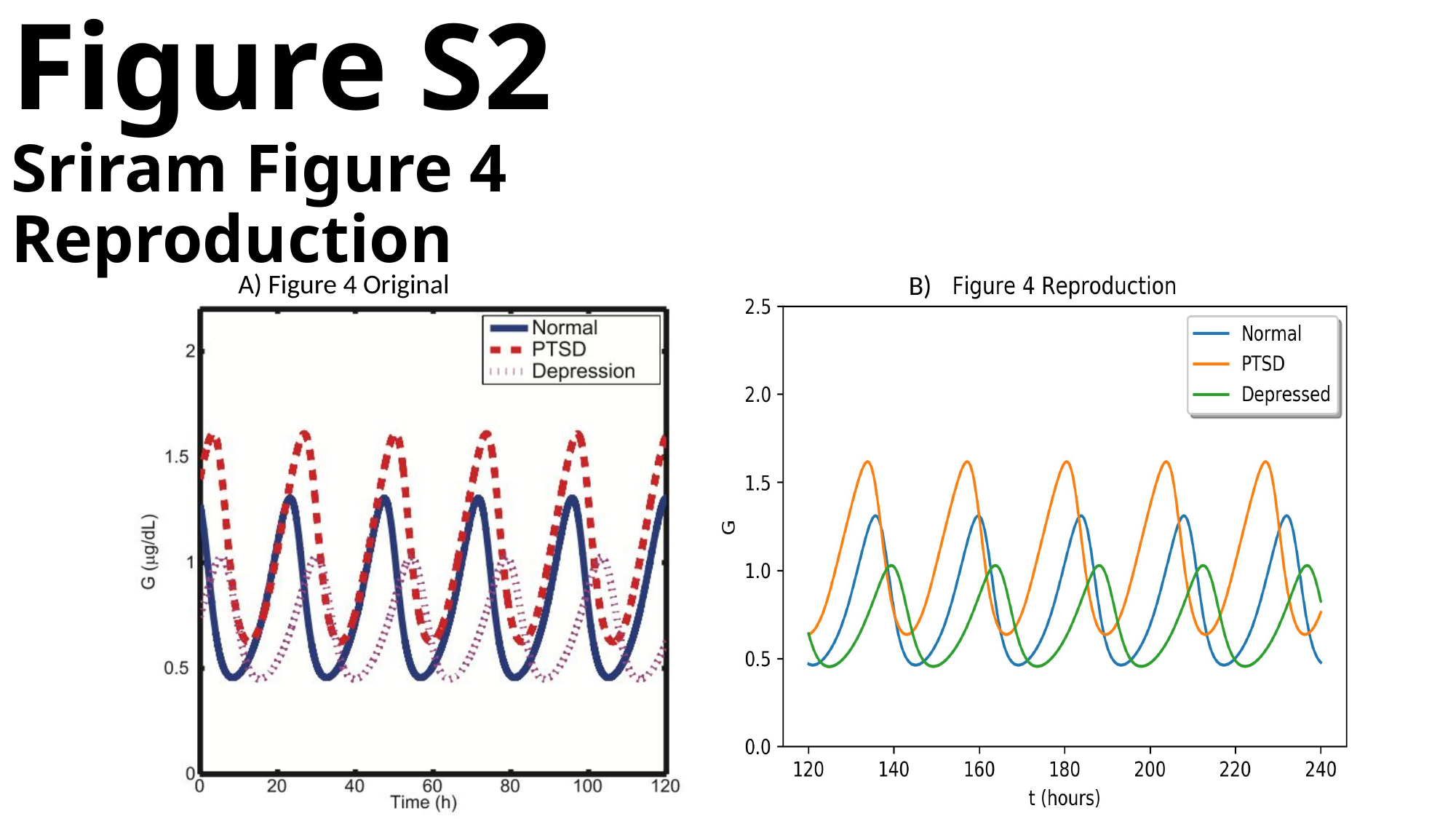

# Figure S2 Sriram Figure 4 Reproduction
A) Figure 4 Original
B)

## Slide 4
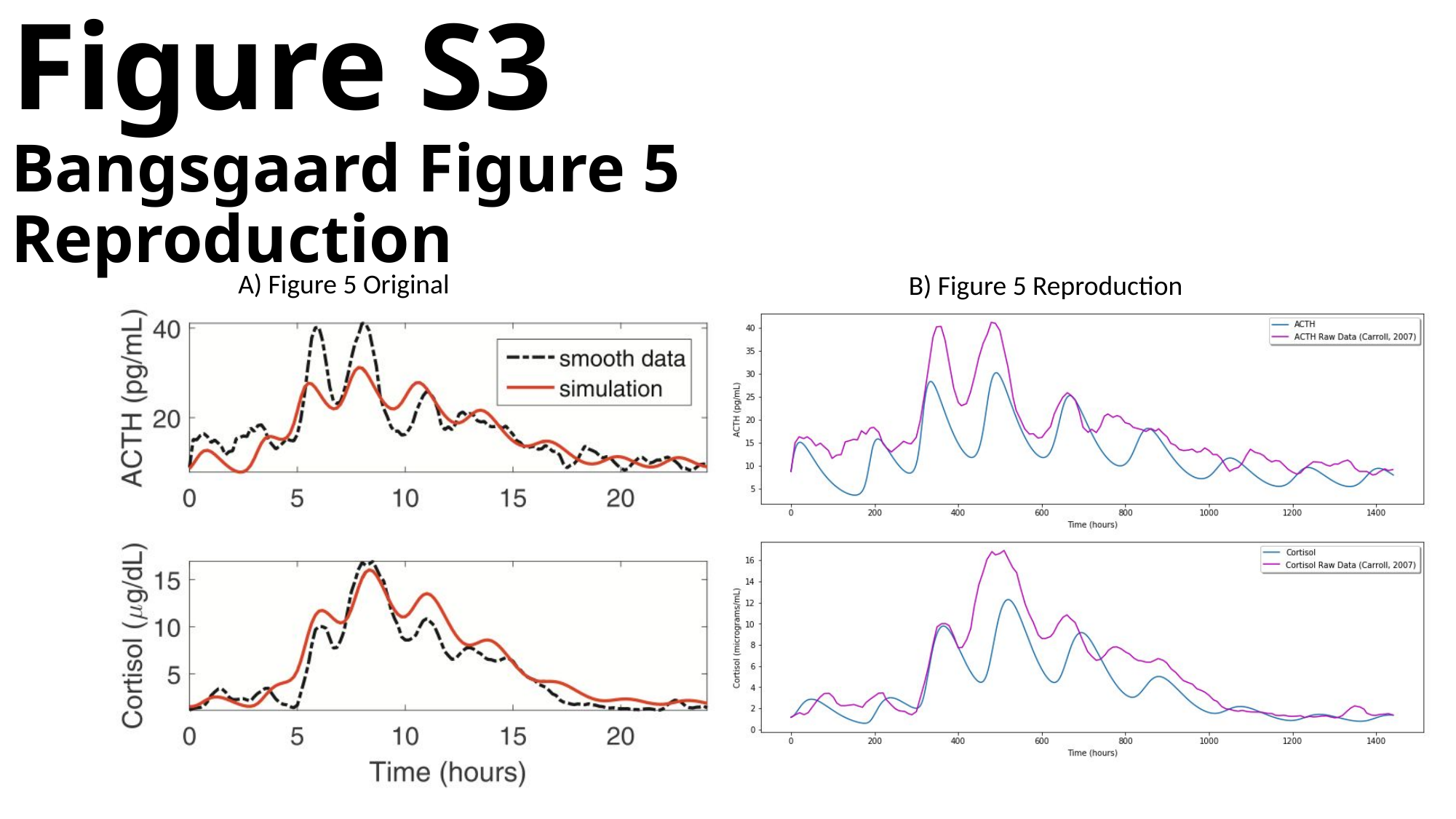

# Figure S3 Bangsgaard Figure 5 Reproduction
A) Figure 5 Original
B) Figure 5 Reproduction

## Slide 5
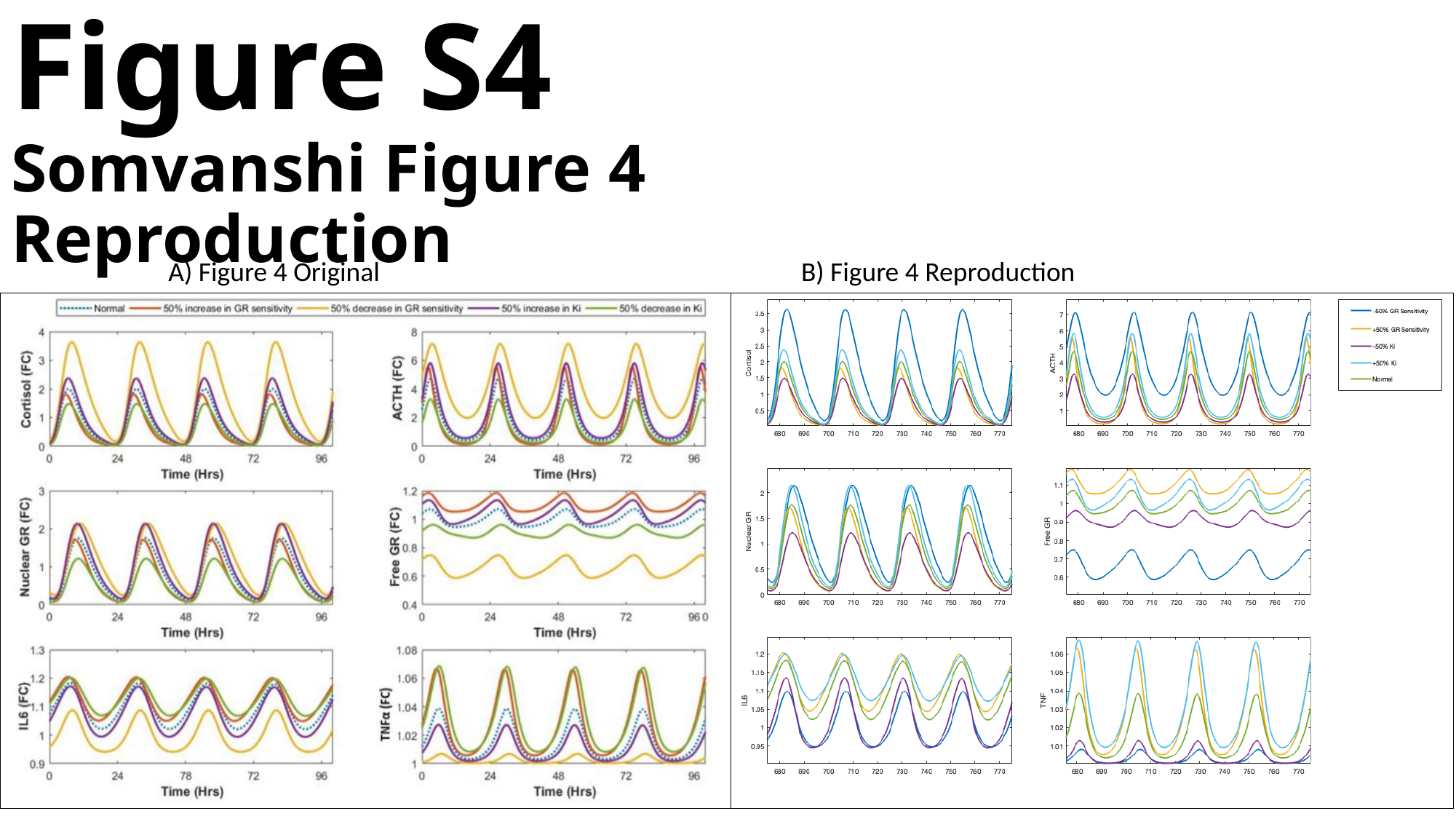

# Figure S4 Somvanshi Figure 4 Reproduction
A) Figure 4 Original
B) Figure 4 Reproduction

## Slide 6
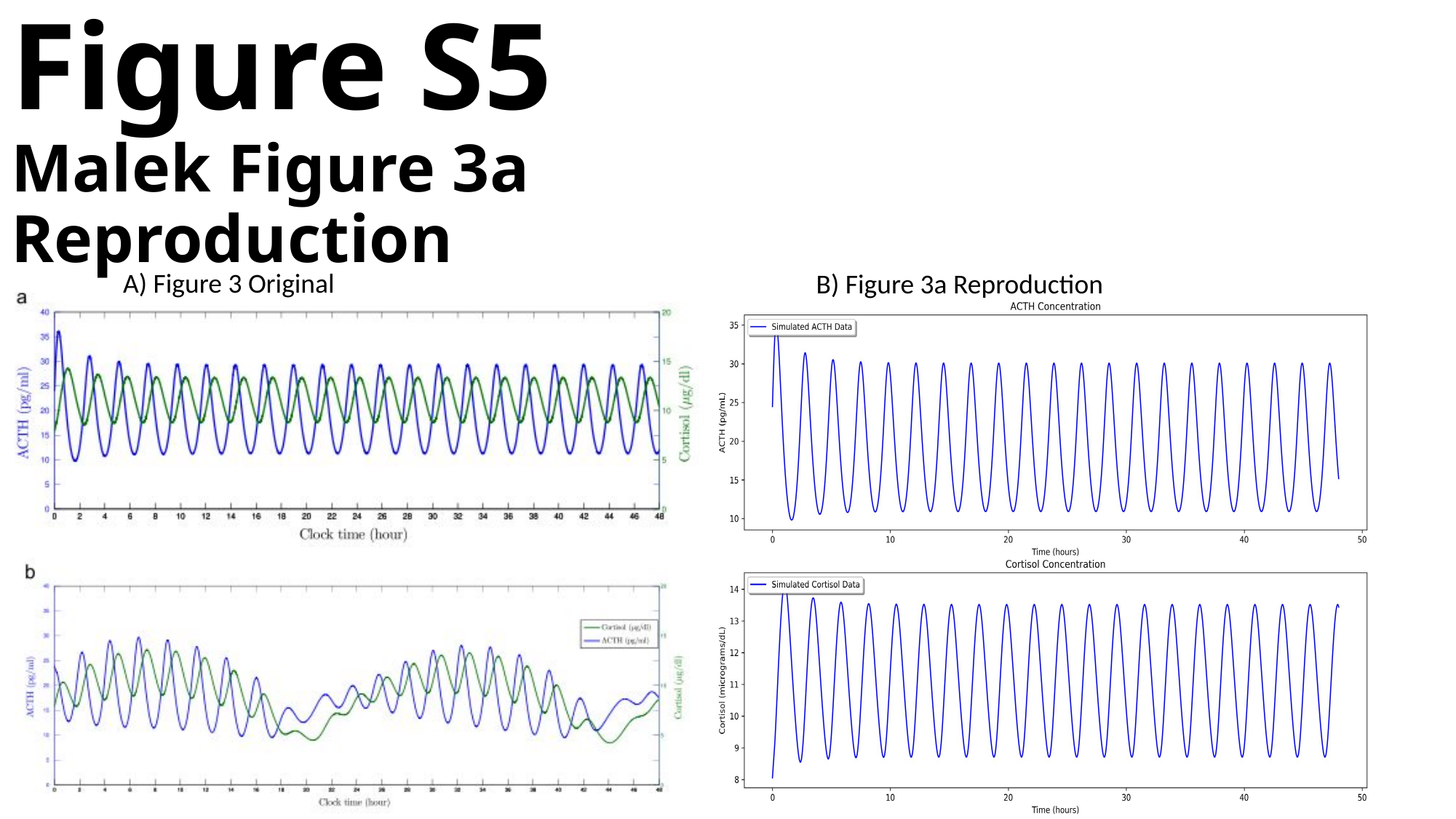

# Figure S5 Malek Figure 3a Reproduction
A) Figure 3 Original
B) Figure 3a Reproduction

## Slide 7
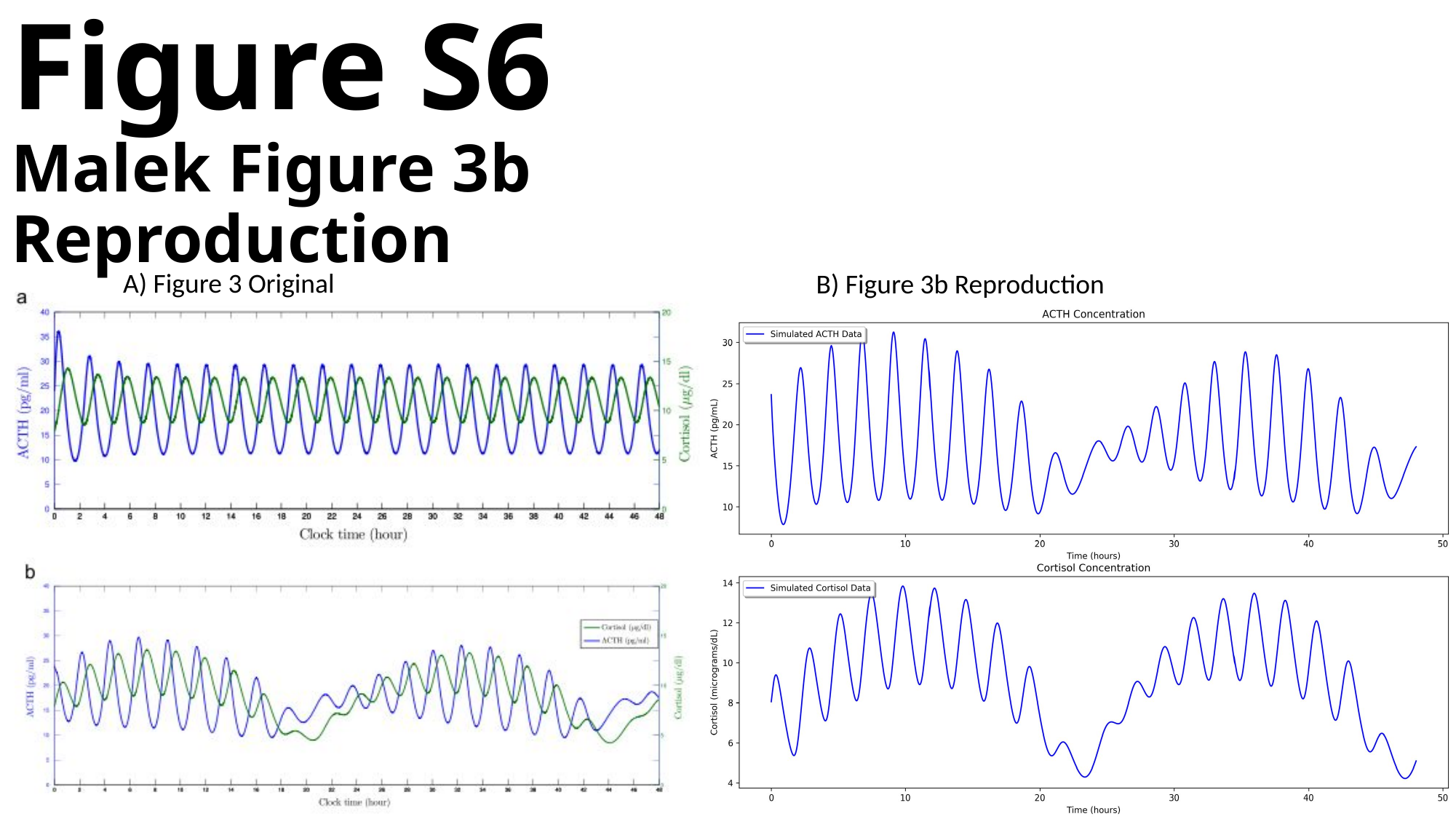

# Figure S6 Malek Figure 3b Reproduction
A) Figure 3 Original
B) Figure 3b Reproduction

## Slide 8
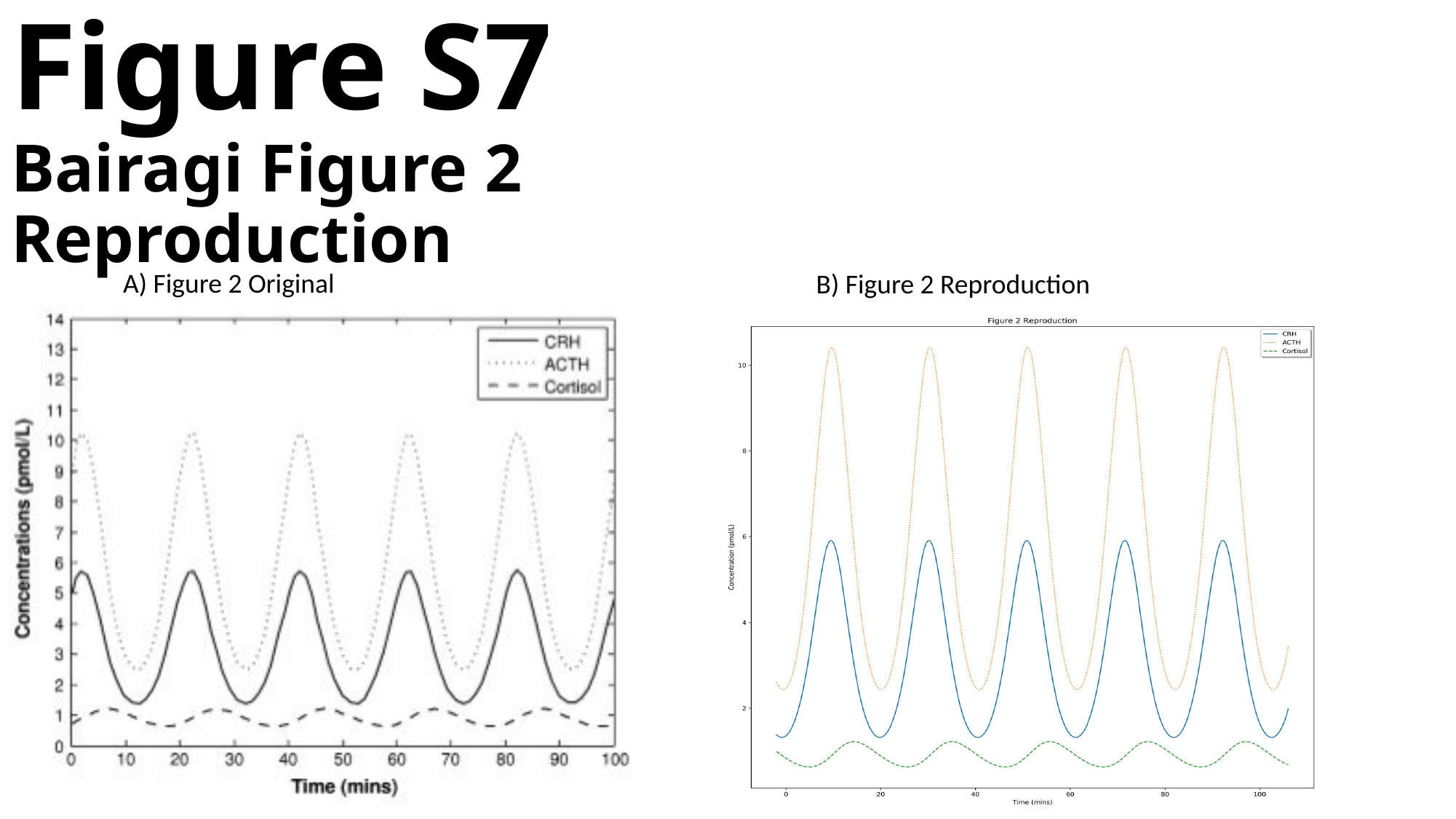

# Figure S7 Bairagi Figure 2 Reproduction
A) Figure 2 Original
B) Figure 2 Reproduction

## Slide 9
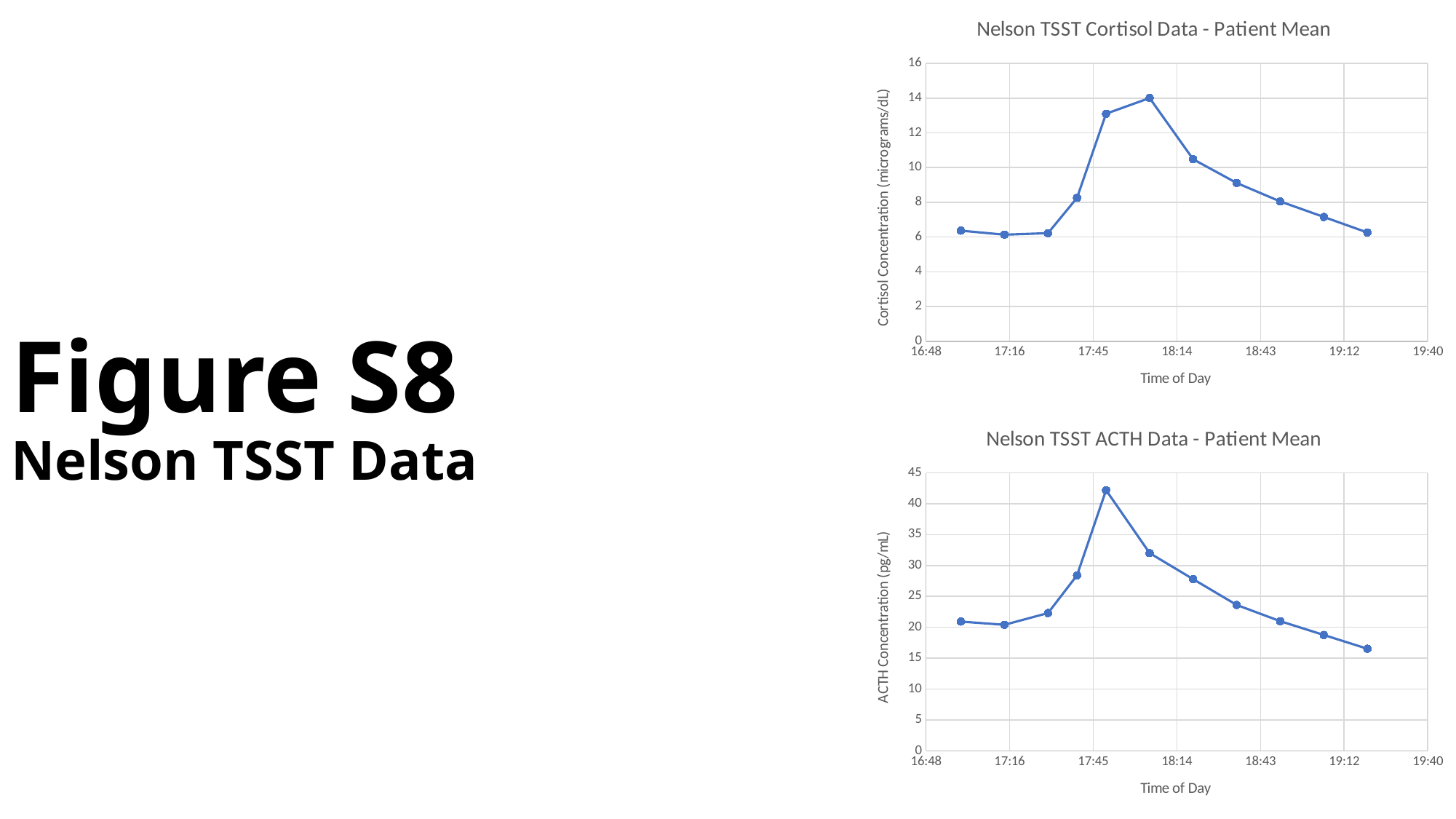

### Chart: Nelson TSST Cortisol Data - Patient Mean
| Category | |
|---|---|# Figure S8
Nelson TSST Data
### Chart: Nelson TSST ACTH Data - Patient Mean
| Category | |
|---|---|

## Slide 10
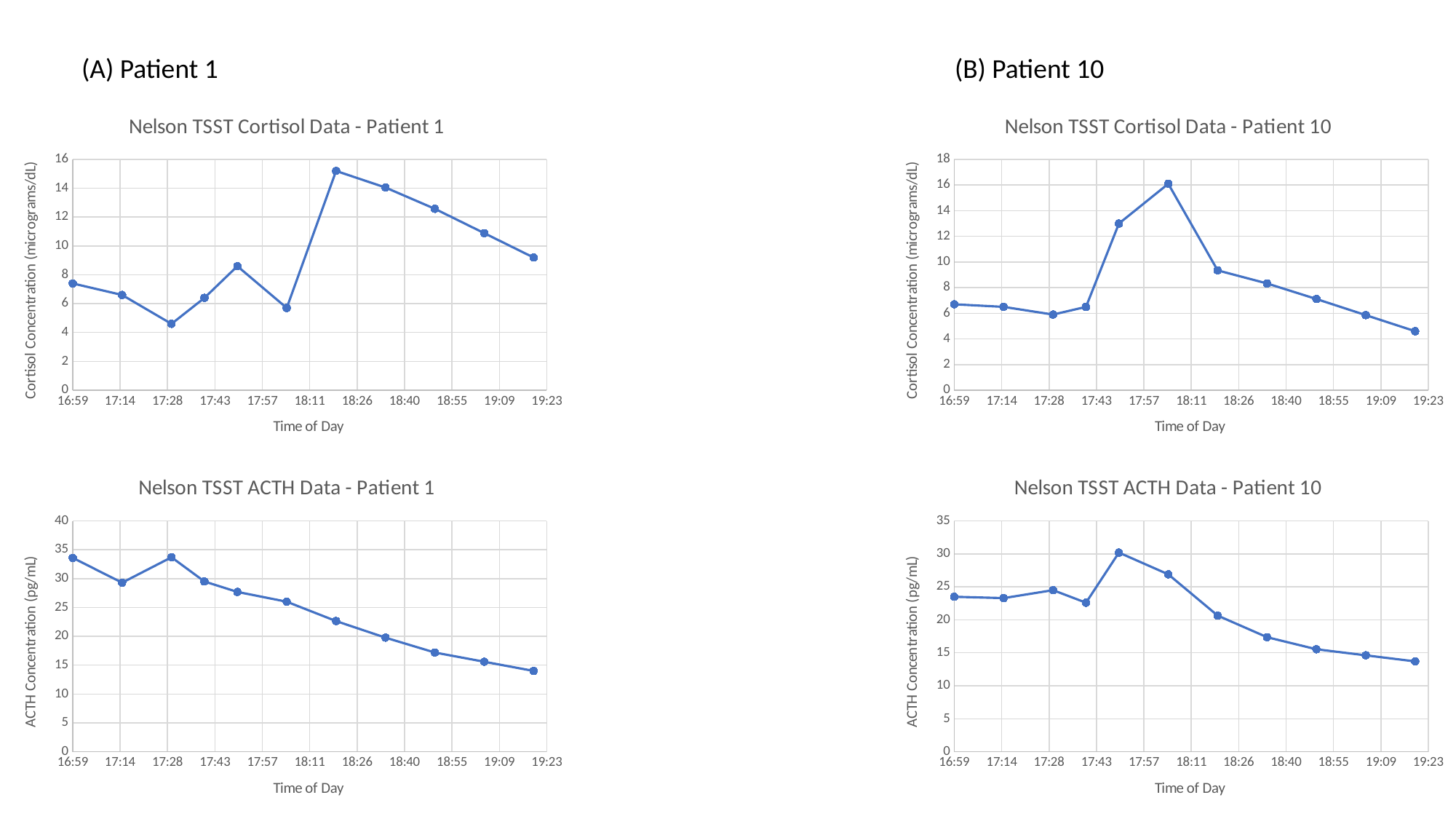

(A) Patient 1
(B) Patient 10
### Chart: Nelson TSST Cortisol Data - Patient 1
| Category | |
|---|---|
### Chart: Nelson TSST Cortisol Data - Patient 10
| Category | |
|---|---|
### Chart: Nelson TSST ACTH Data - Patient 1
| Category | |
|---|---|
### Chart: Nelson TSST ACTH Data - Patient 10
| Category | |
|---|---|

## Slide 11
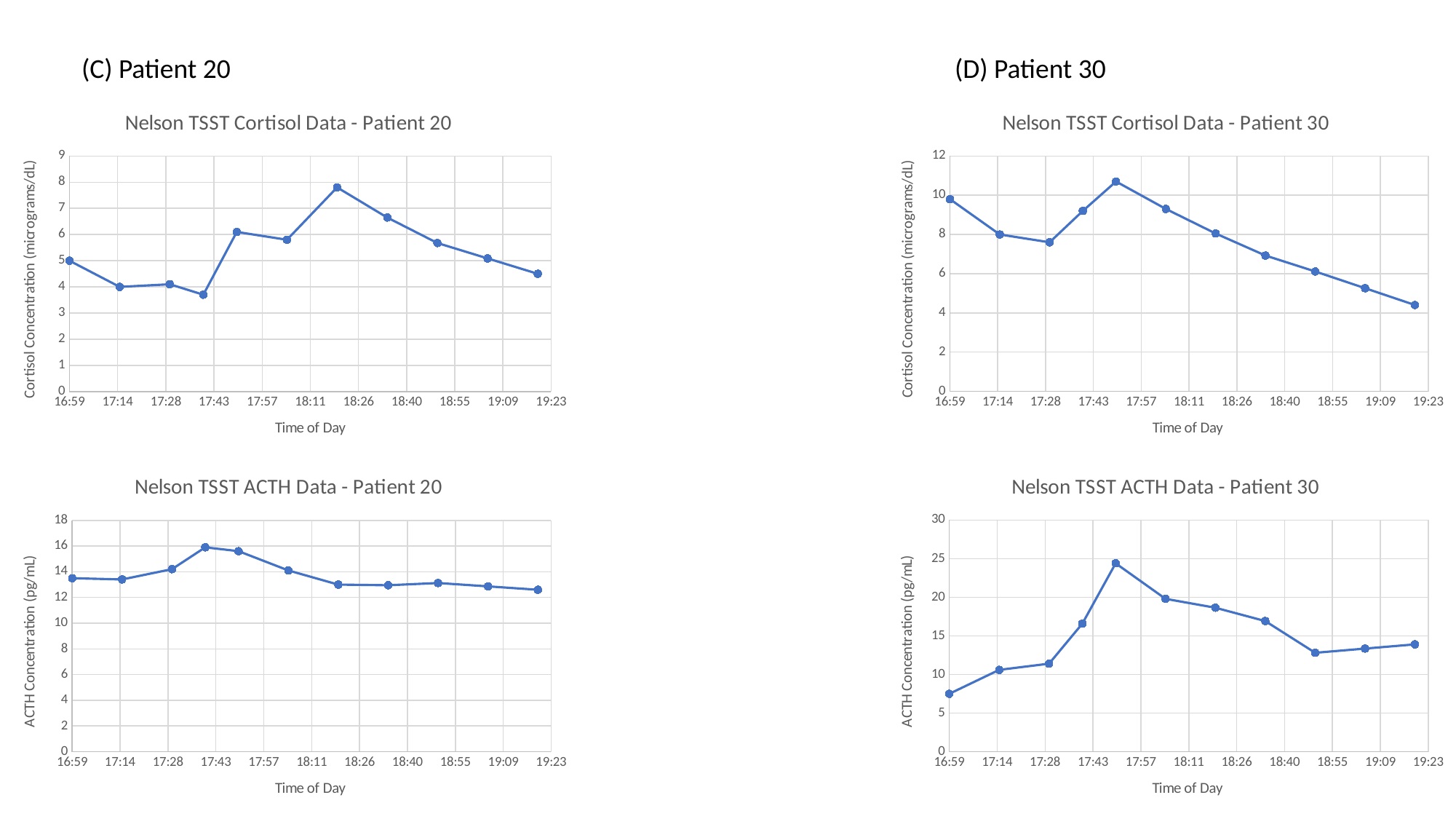

(C) Patient 20
(D) Patient 30
### Chart: Nelson TSST Cortisol Data - Patient 30
| Category | |
|---|---|
### Chart: Nelson TSST Cortisol Data - Patient 20
| Category | |
|---|---|
### Chart: Nelson TSST ACTH Data - Patient 30
| Category | |
|---|---|
### Chart: Nelson TSST ACTH Data - Patient 20
| Category | |
|---|---|

## Slide 12
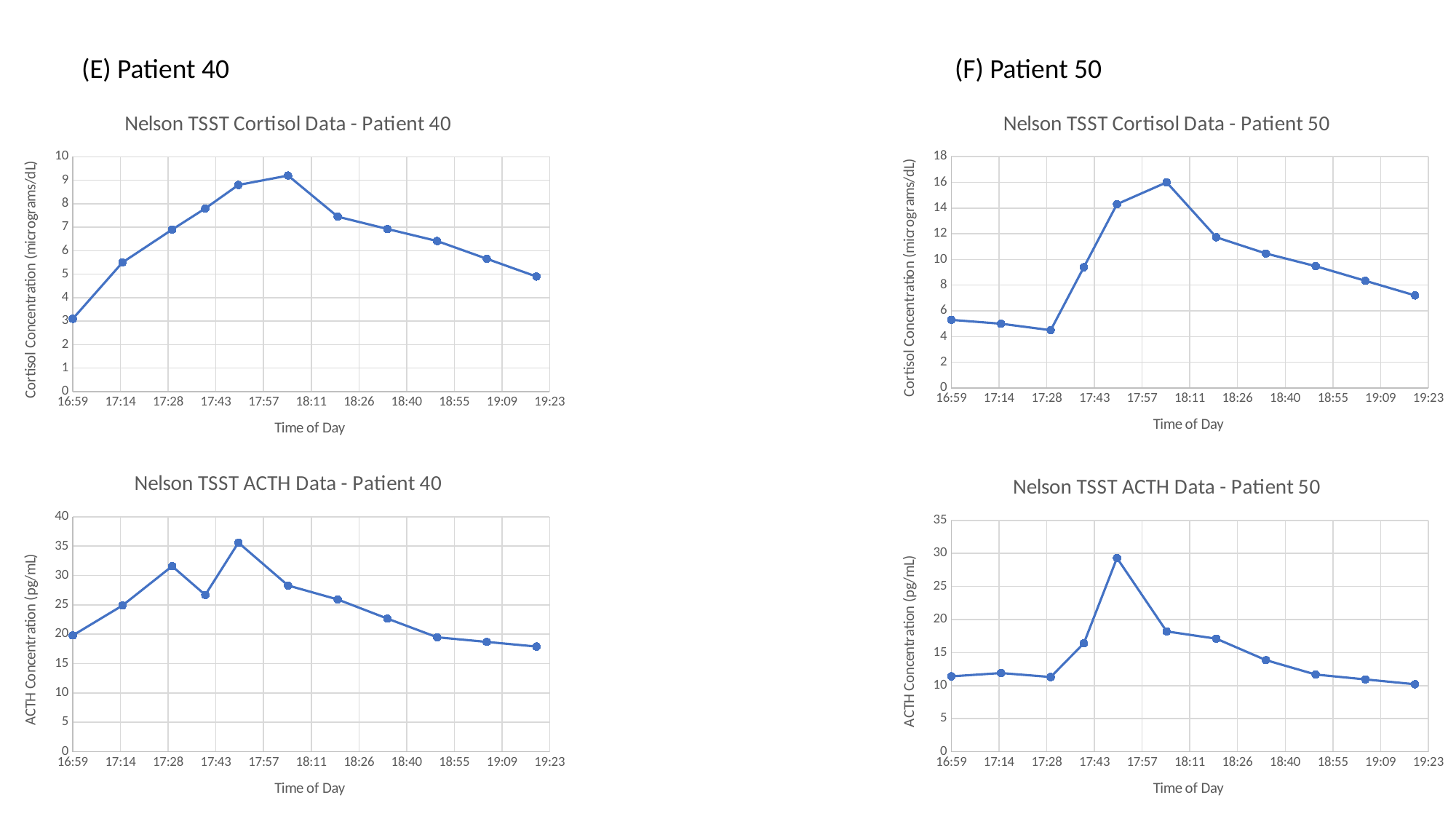

(E) Patient 40
(F) Patient 50
### Chart: Nelson TSST Cortisol Data - Patient 50
| Category | |
|---|---|
### Chart: Nelson TSST Cortisol Data - Patient 40
| Category | |
|---|---|
### Chart: Nelson TSST ACTH Data - Patient 40
| Category | |
|---|---|
### Chart: Nelson TSST ACTH Data - Patient 50
| Category | |
|---|---|

## Slide 13
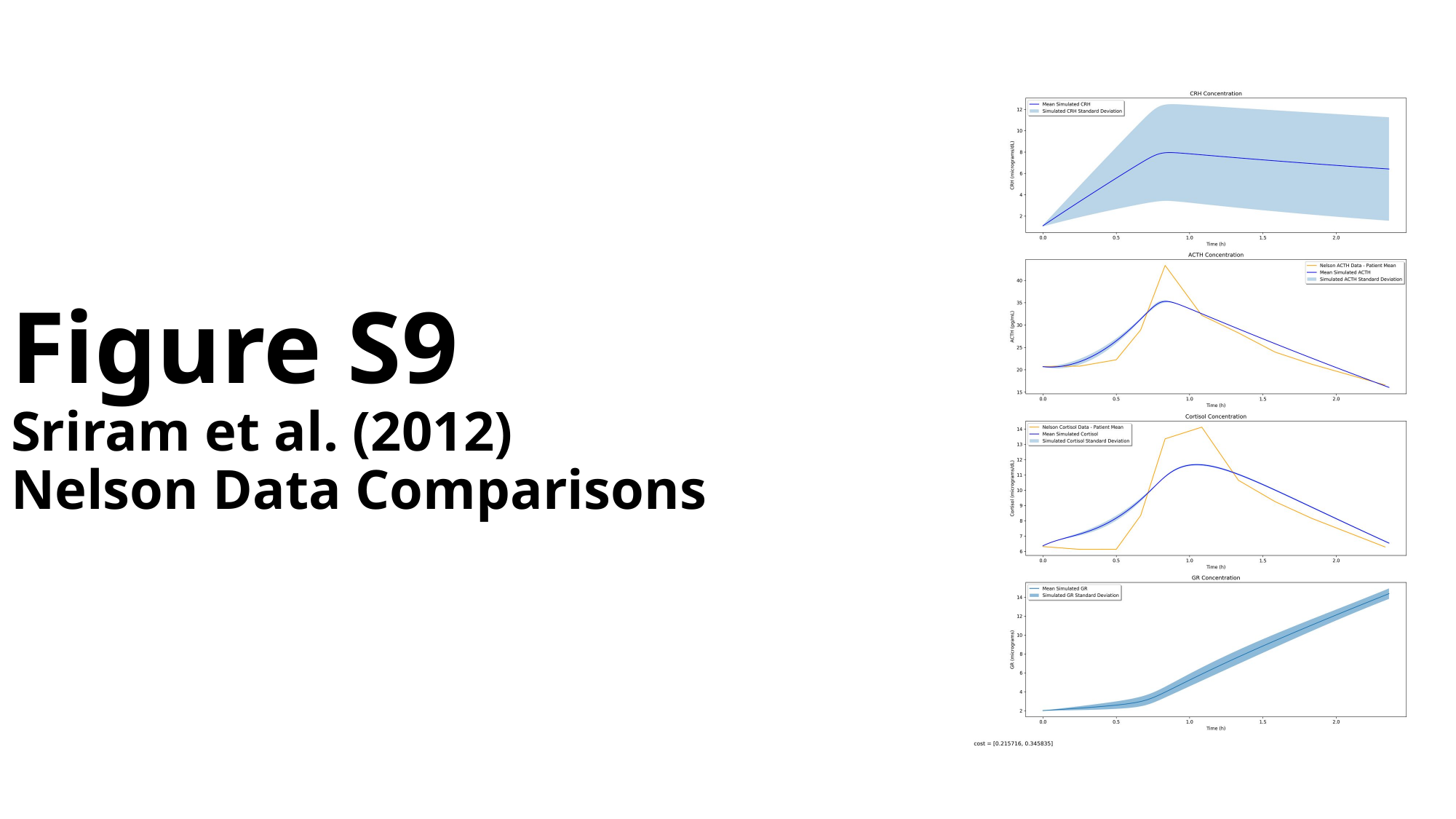

# Figure S9 Sriram et al. (2012) Nelson Data Comparisons

## Slide 14
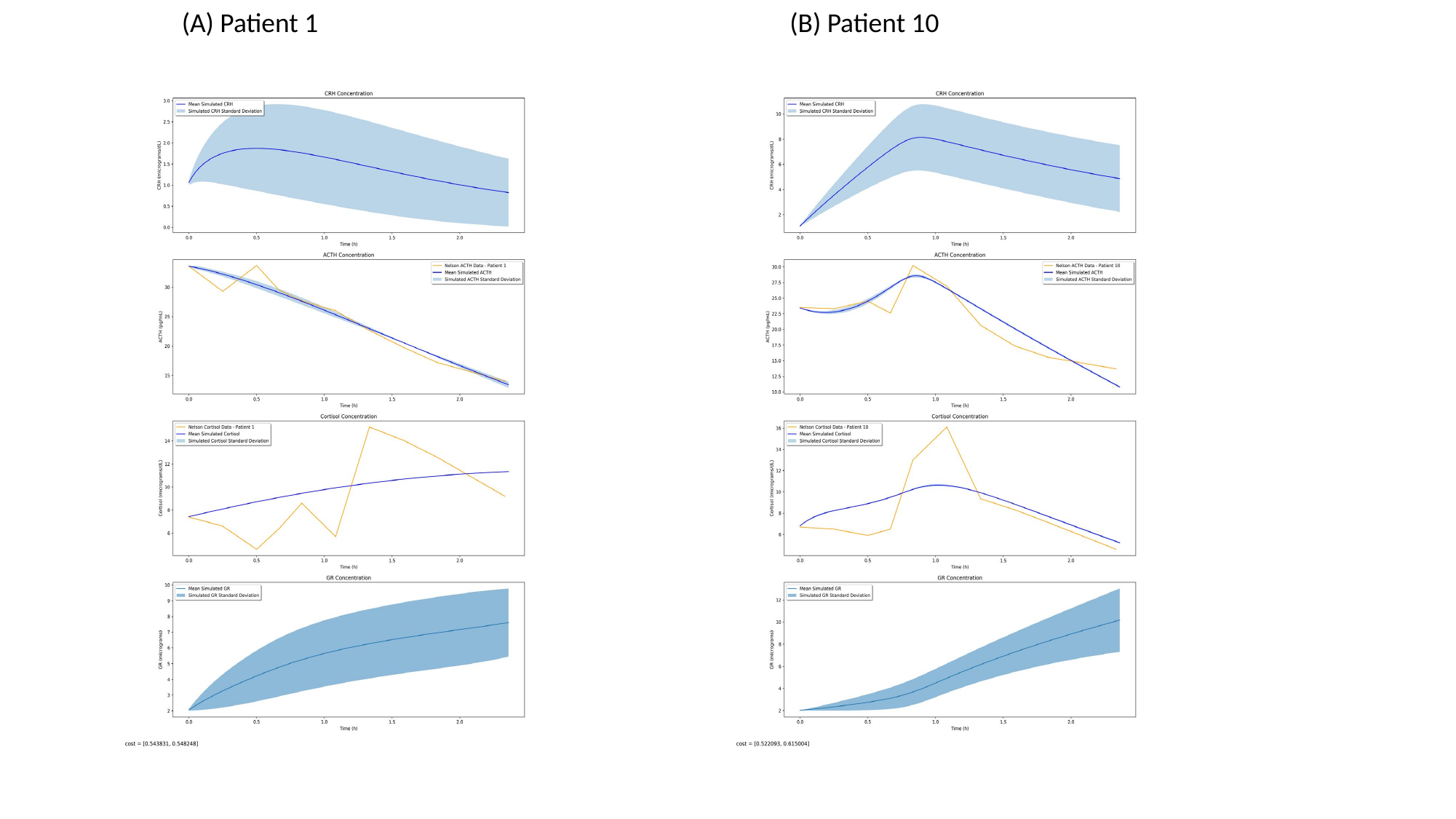

(A) Patient 1
(B) Patient 10

## Slide 15
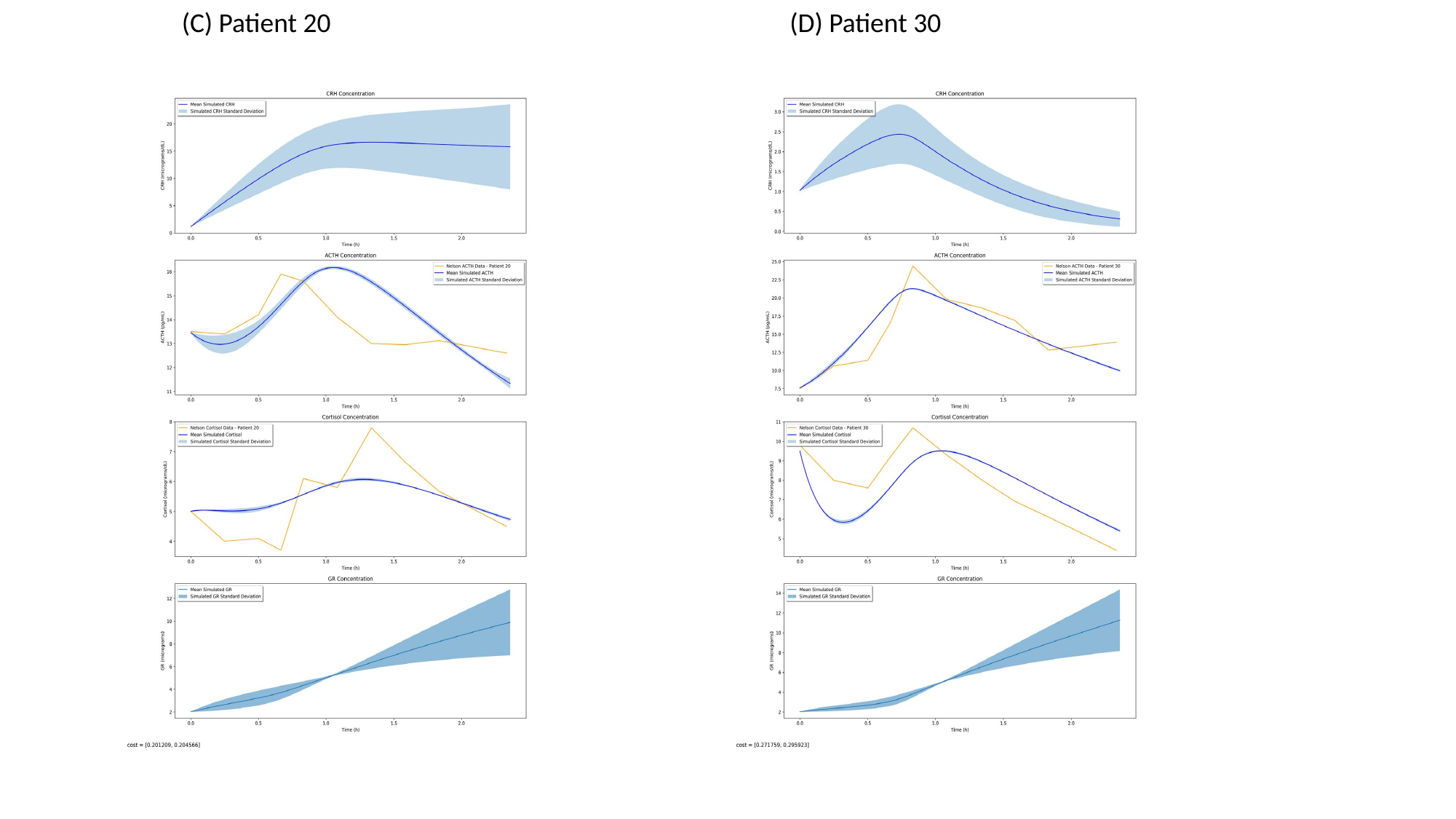

(C) Patient 20
(D) Patient 30

## Slide 16
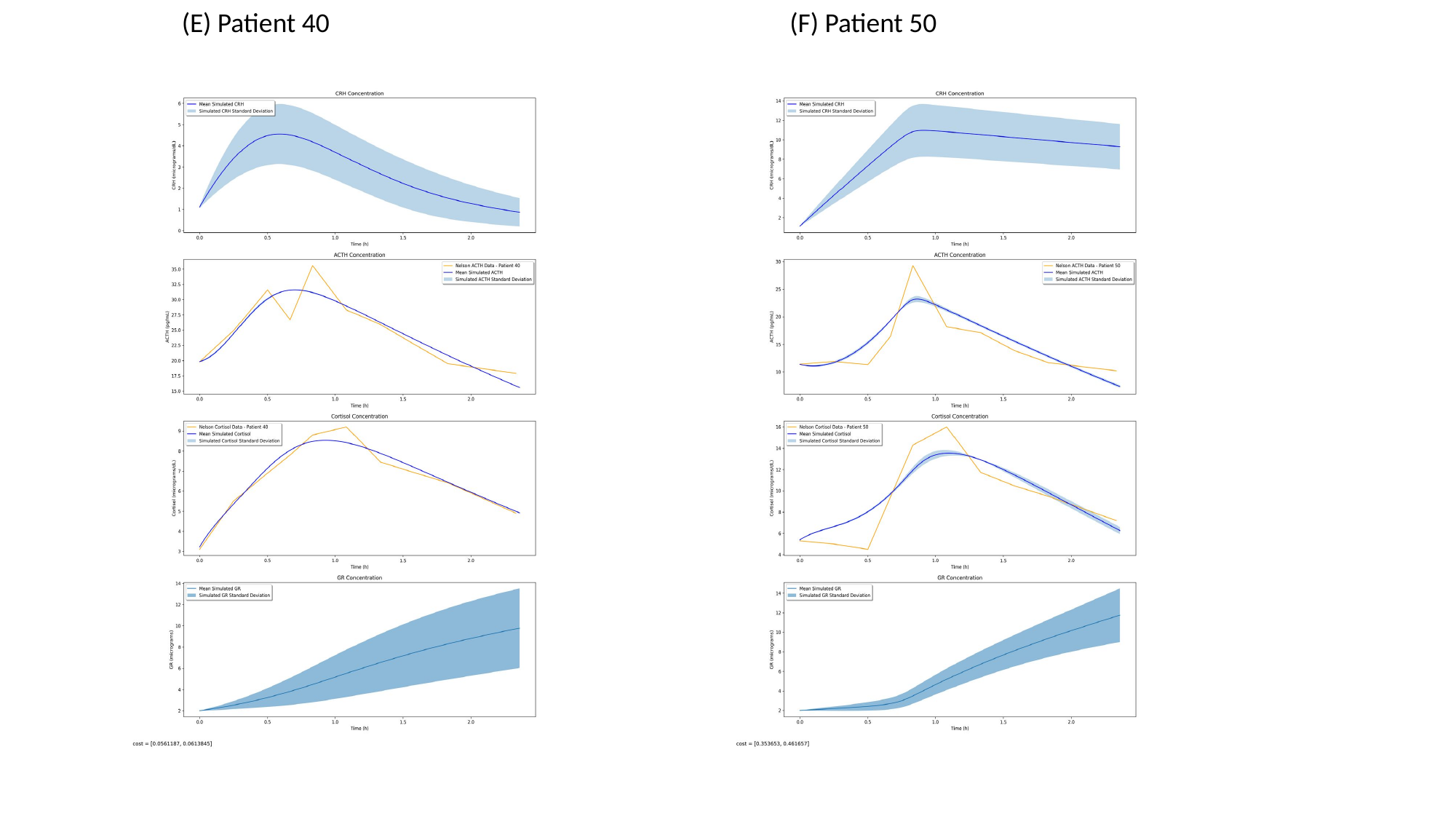

(E) Patient 40
(F) Patient 50

## Slide 17
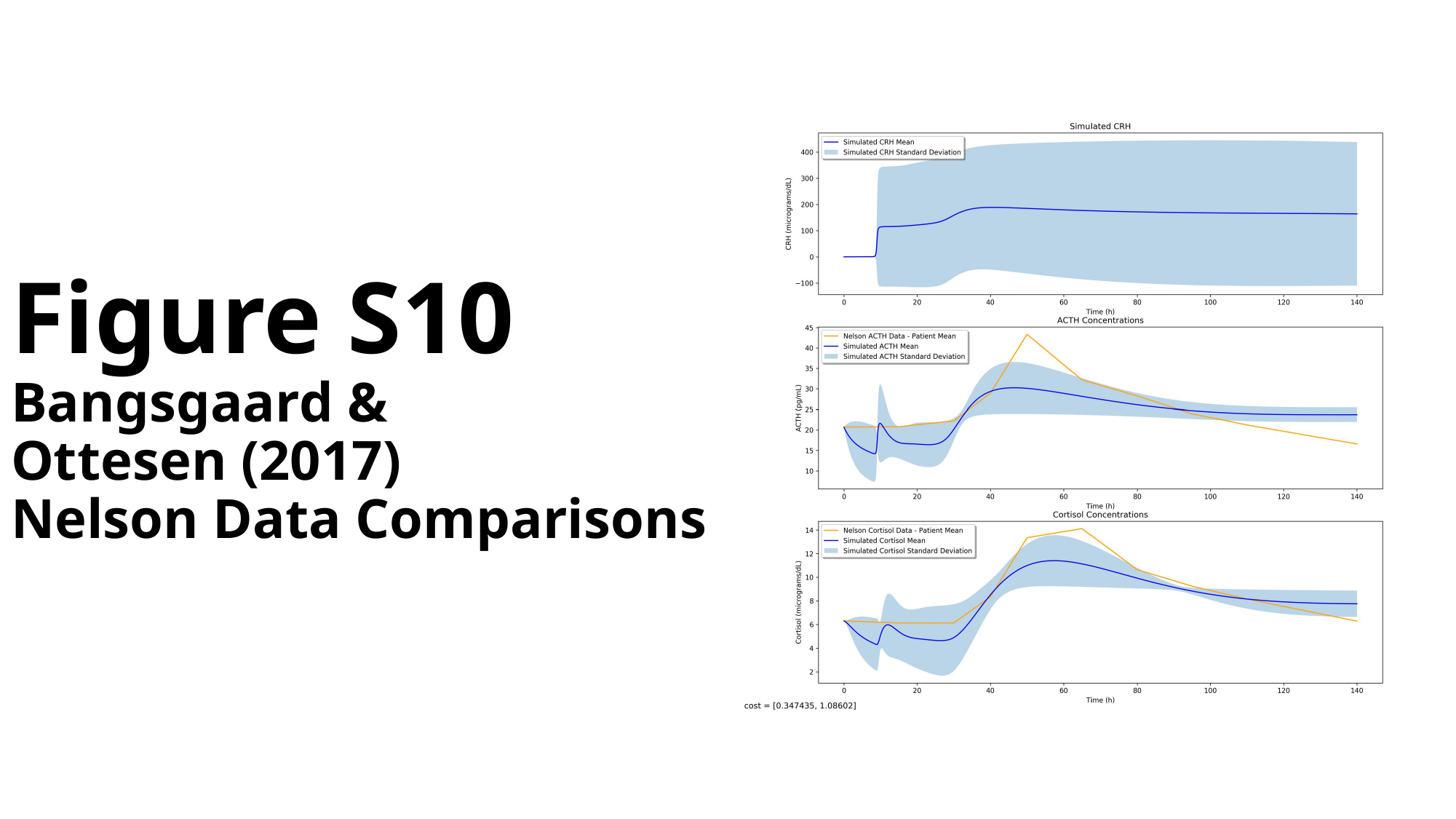

# Figure S10 Bangsgaard & Ottesen (2017) Nelson Data Comparisons

## Slide 18
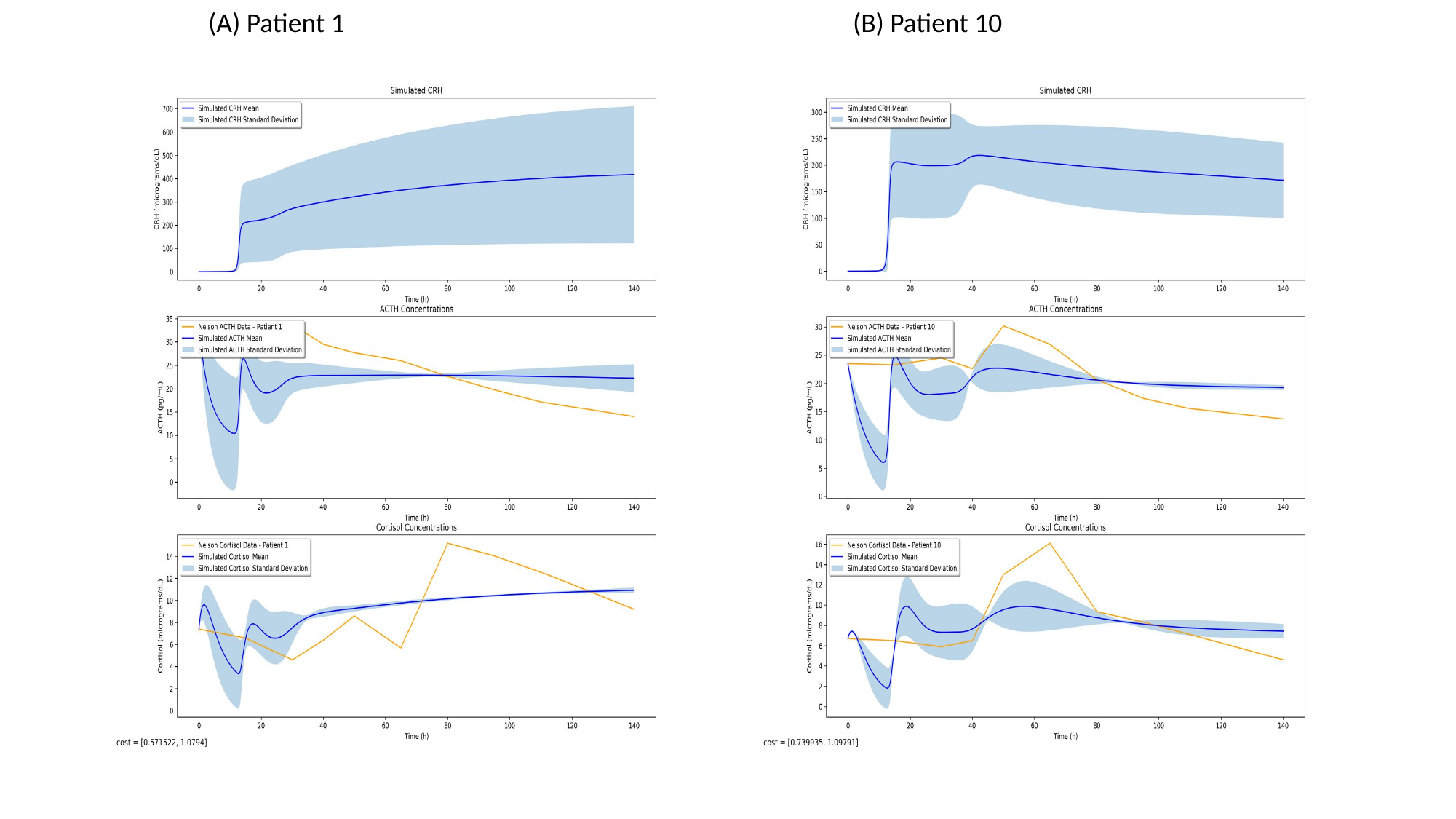

(A) Patient 1
(B) Patient 10

## Slide 19
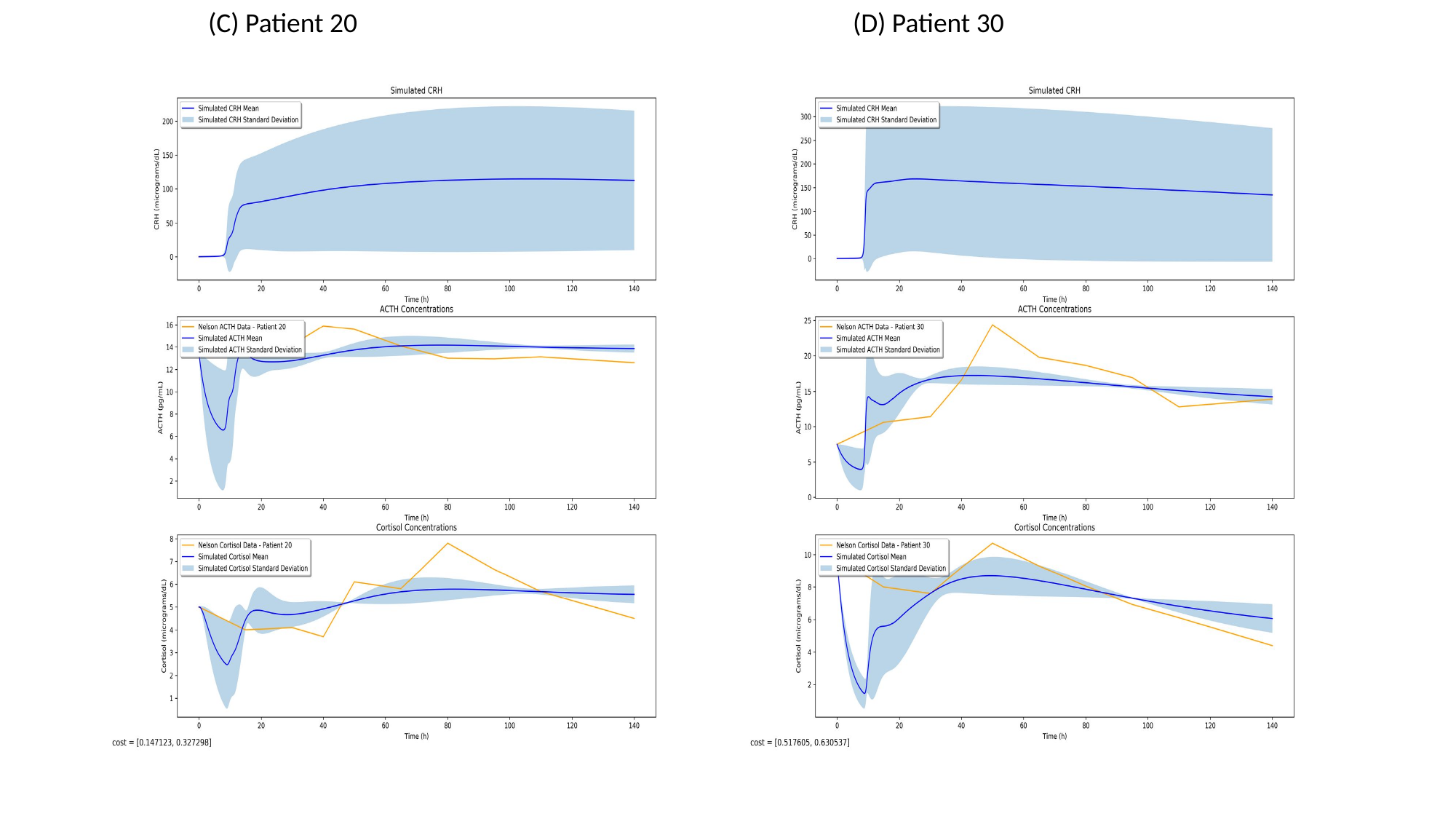

(C) Patient 20
(D) Patient 30

## Slide 20
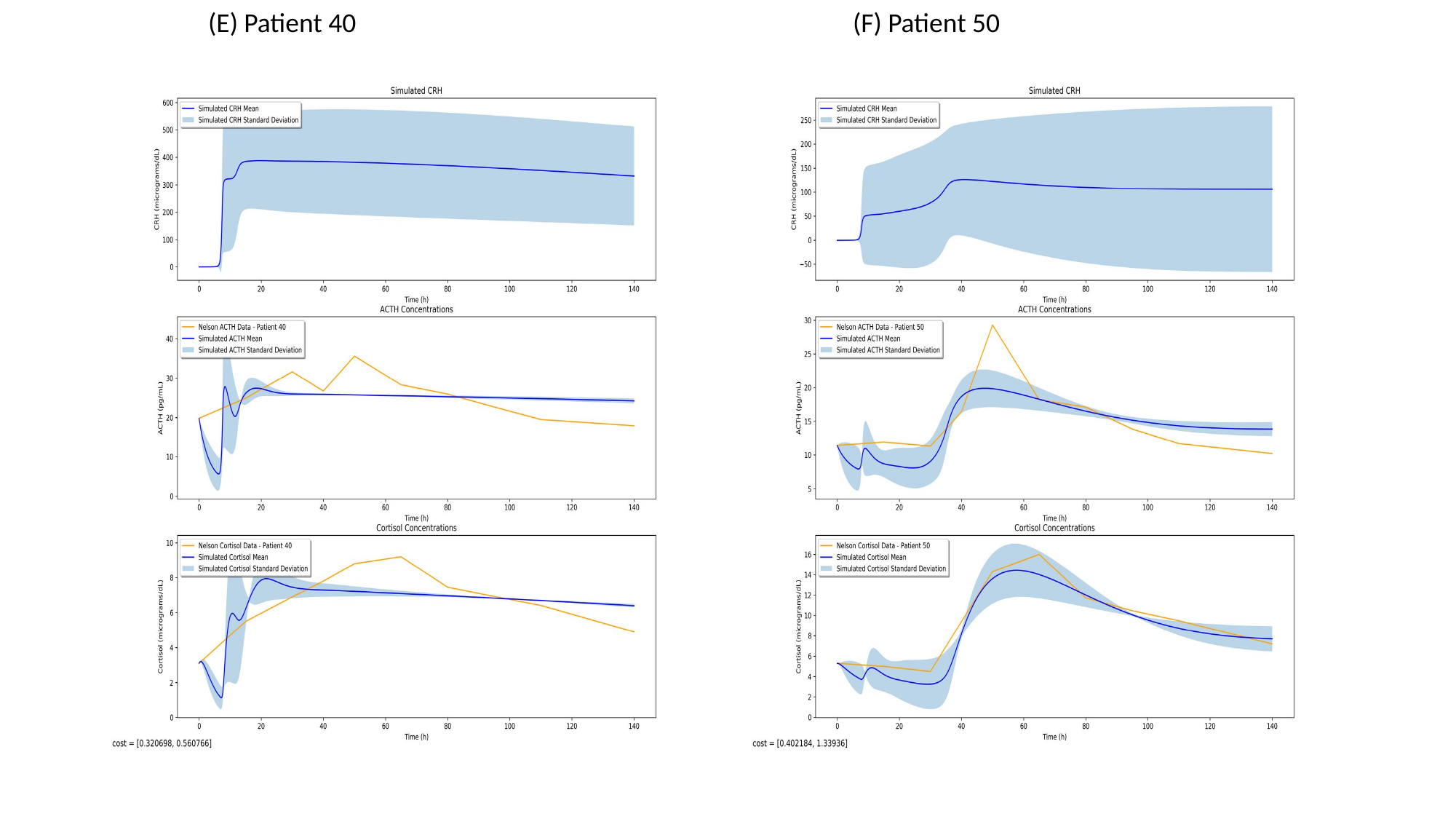

(E) Patient 40
(F) Patient 50

## Slide 21
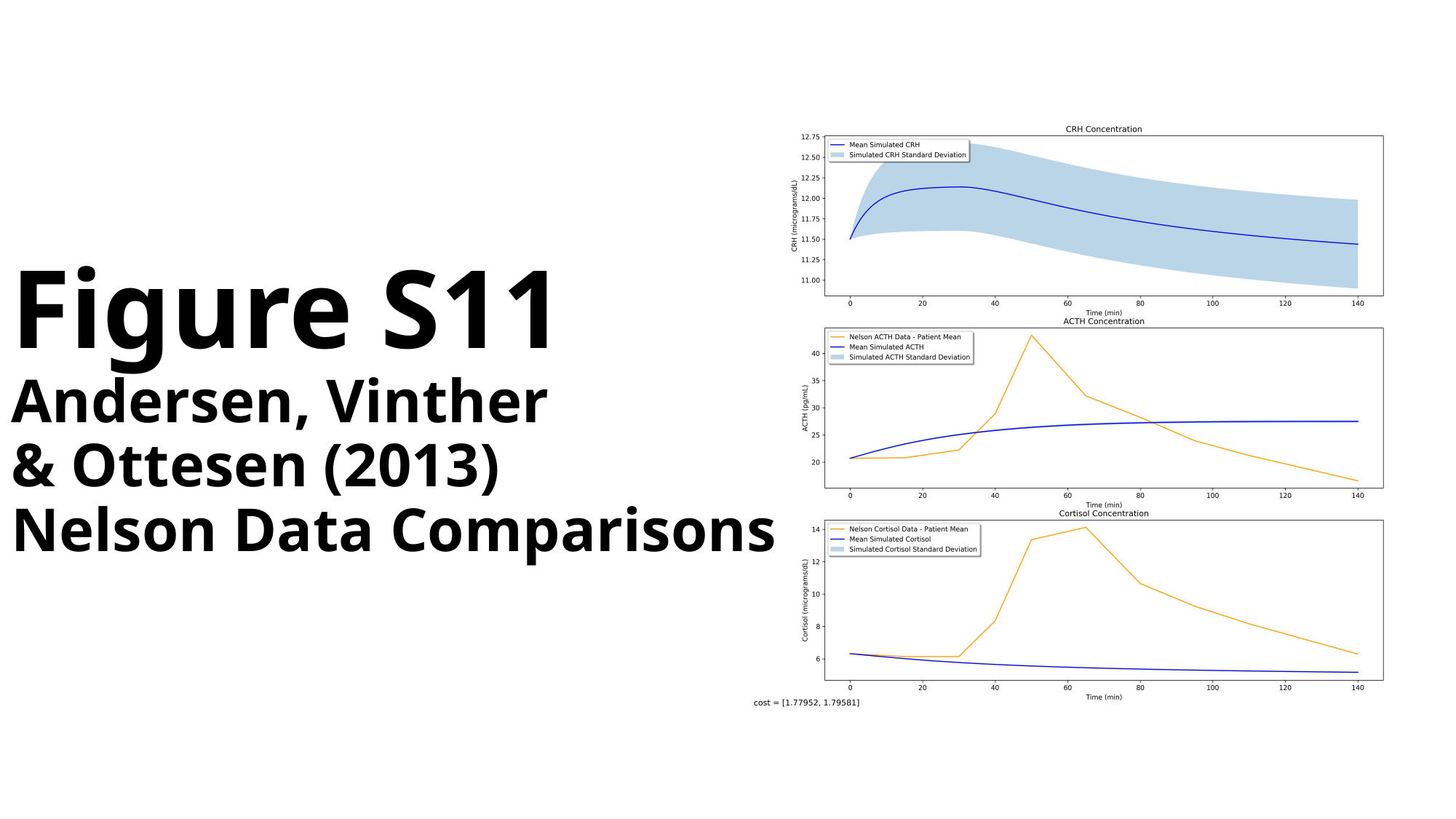

# Figure S11 Andersen, Vinther & Ottesen (2013) Nelson Data Comparisons

## Slide 22
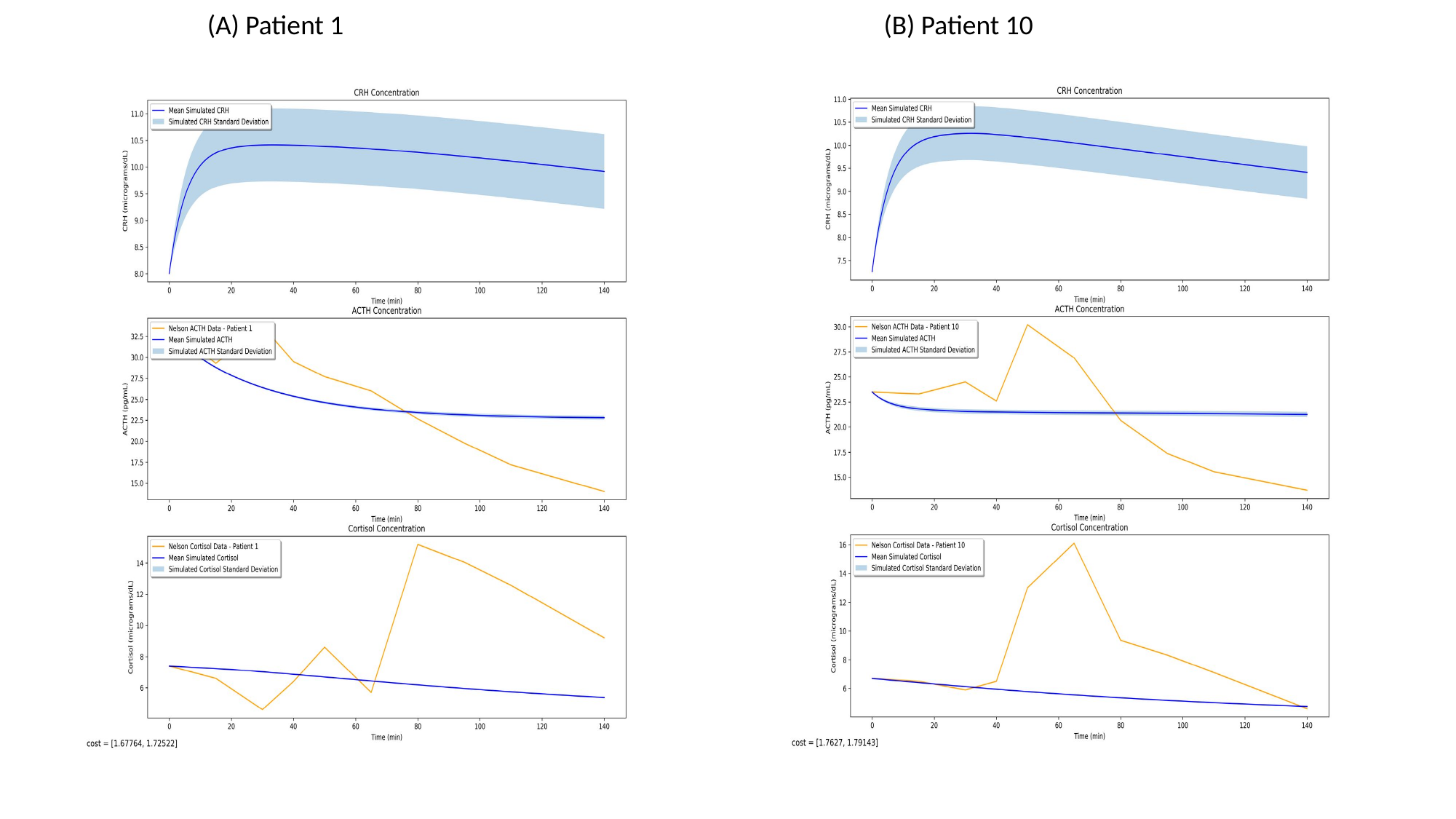

(A) Patient 1
(B) Patient 10

## Slide 23
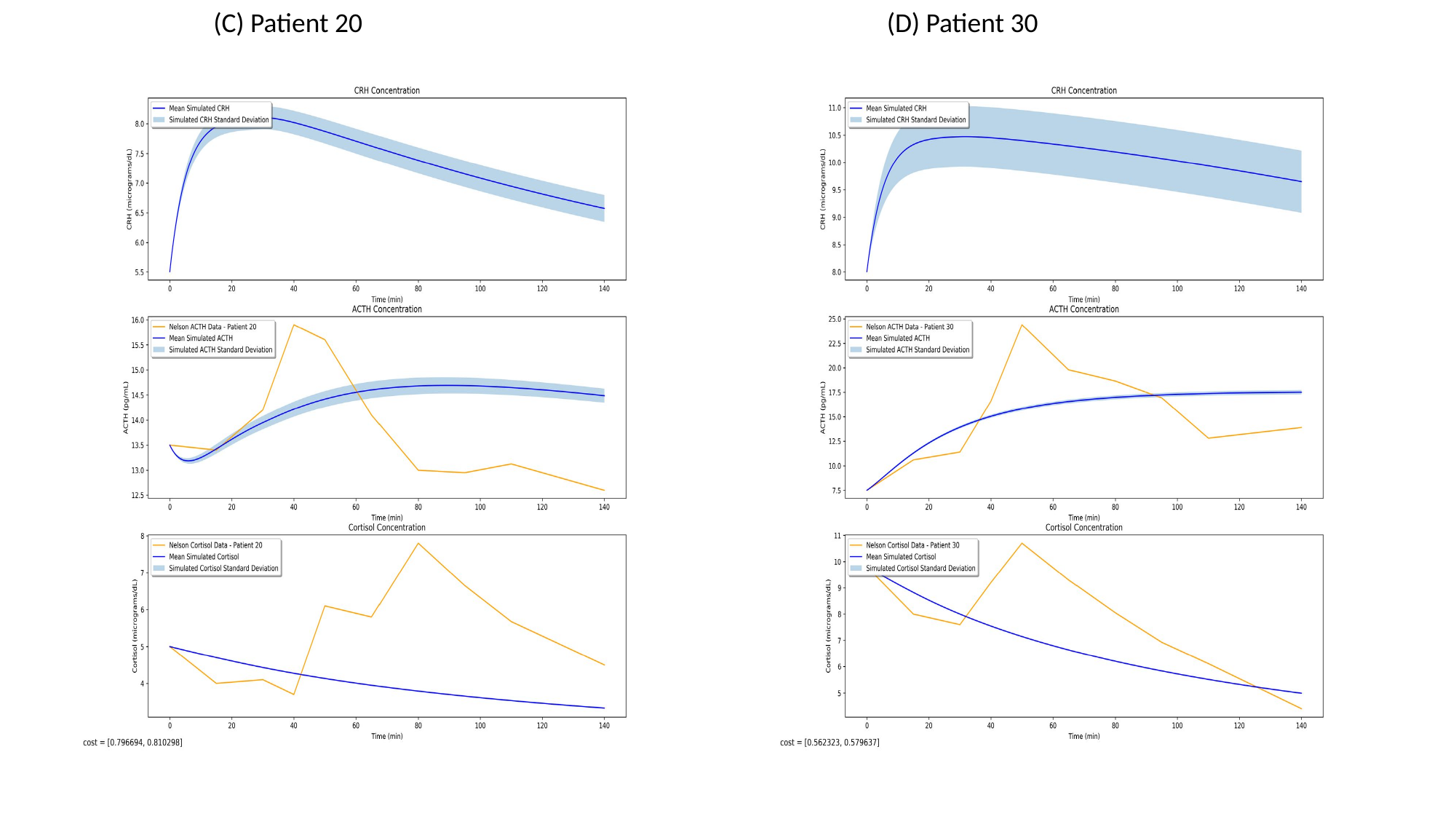

(C) Patient 20
(D) Patient 30

## Slide 24
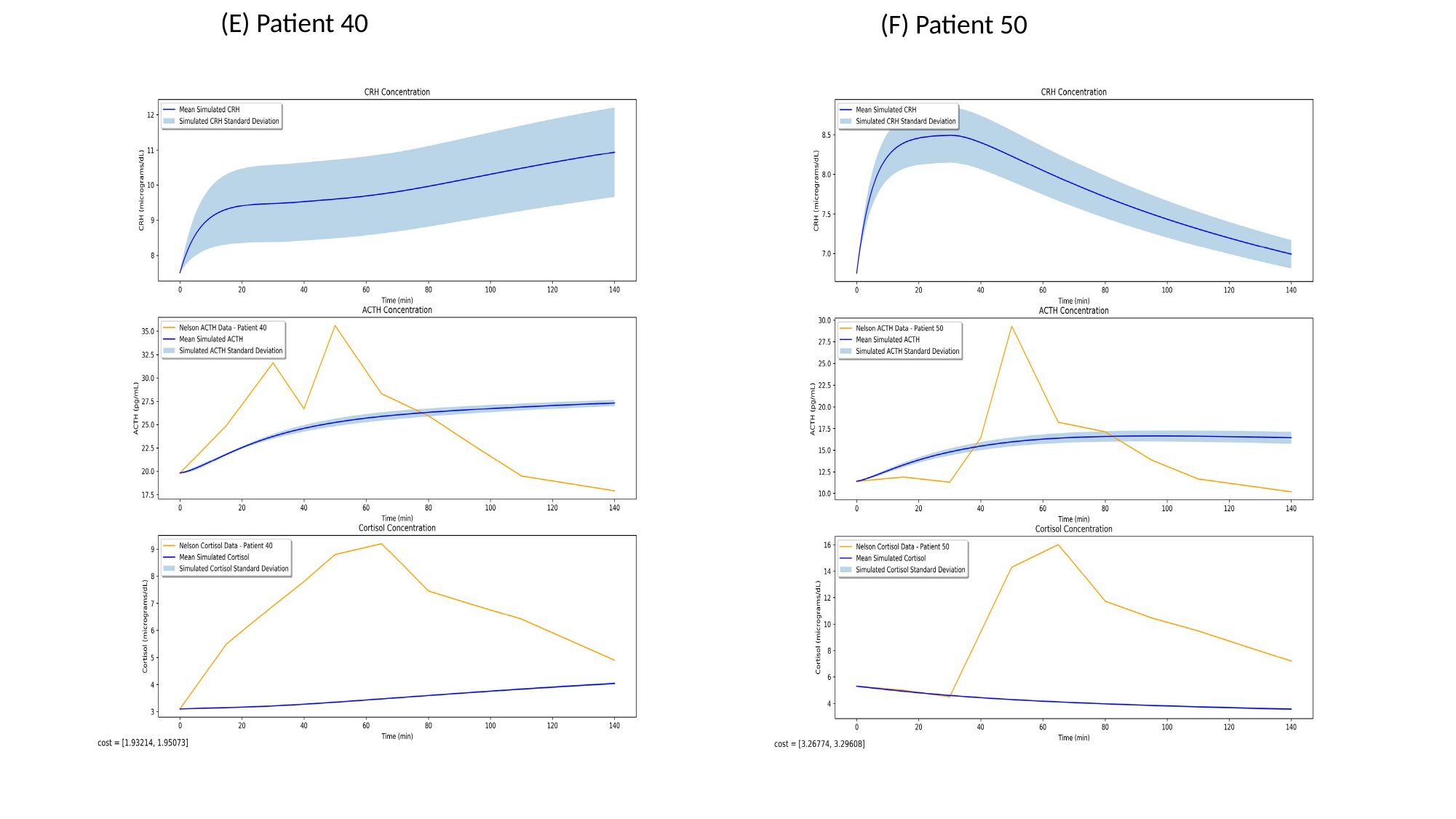

(E) Patient 40
(F) Patient 50

## Slide 25
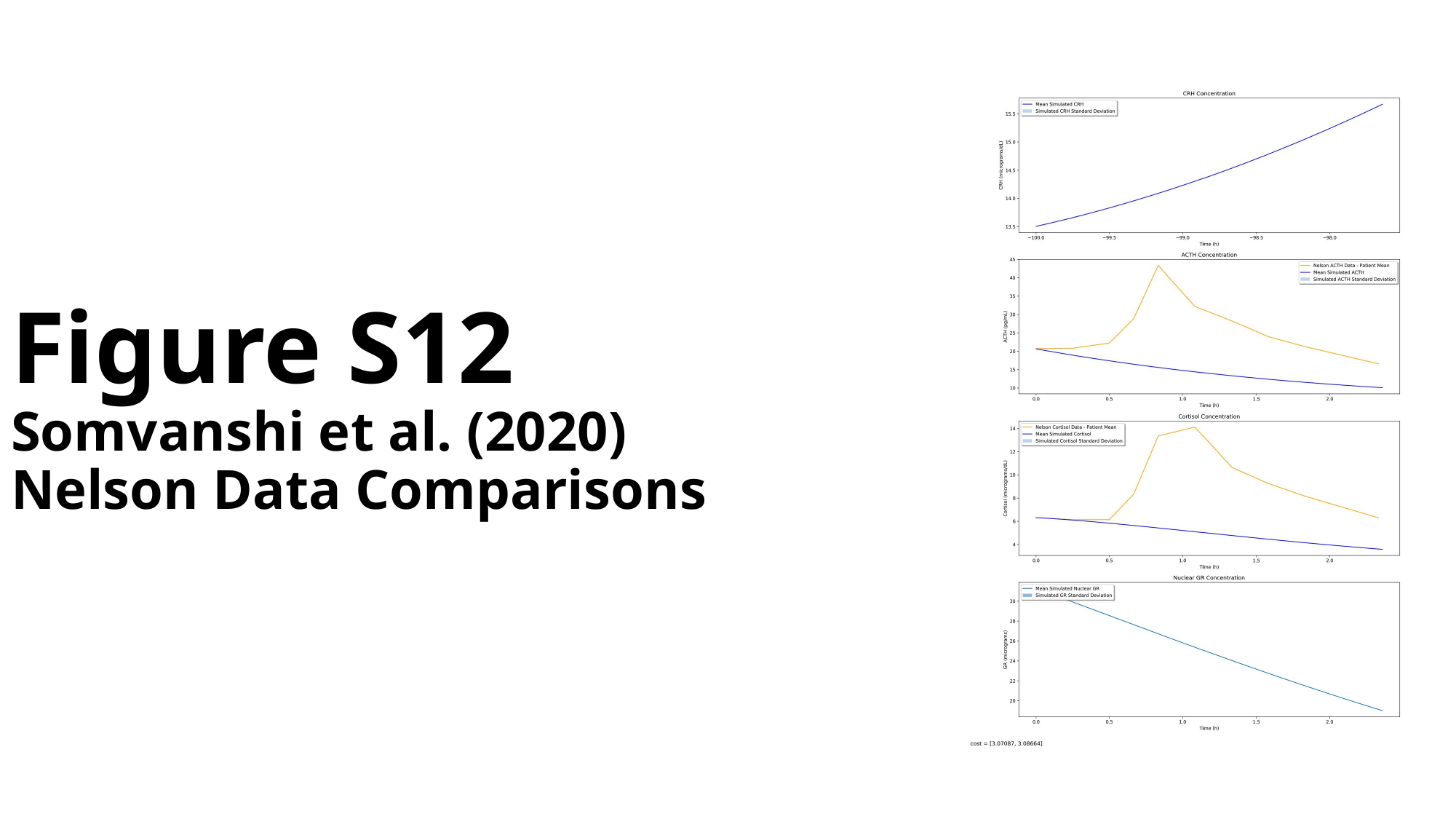

# Figure S12 Somvanshi et al. (2020) Nelson Data Comparisons

## Slide 26
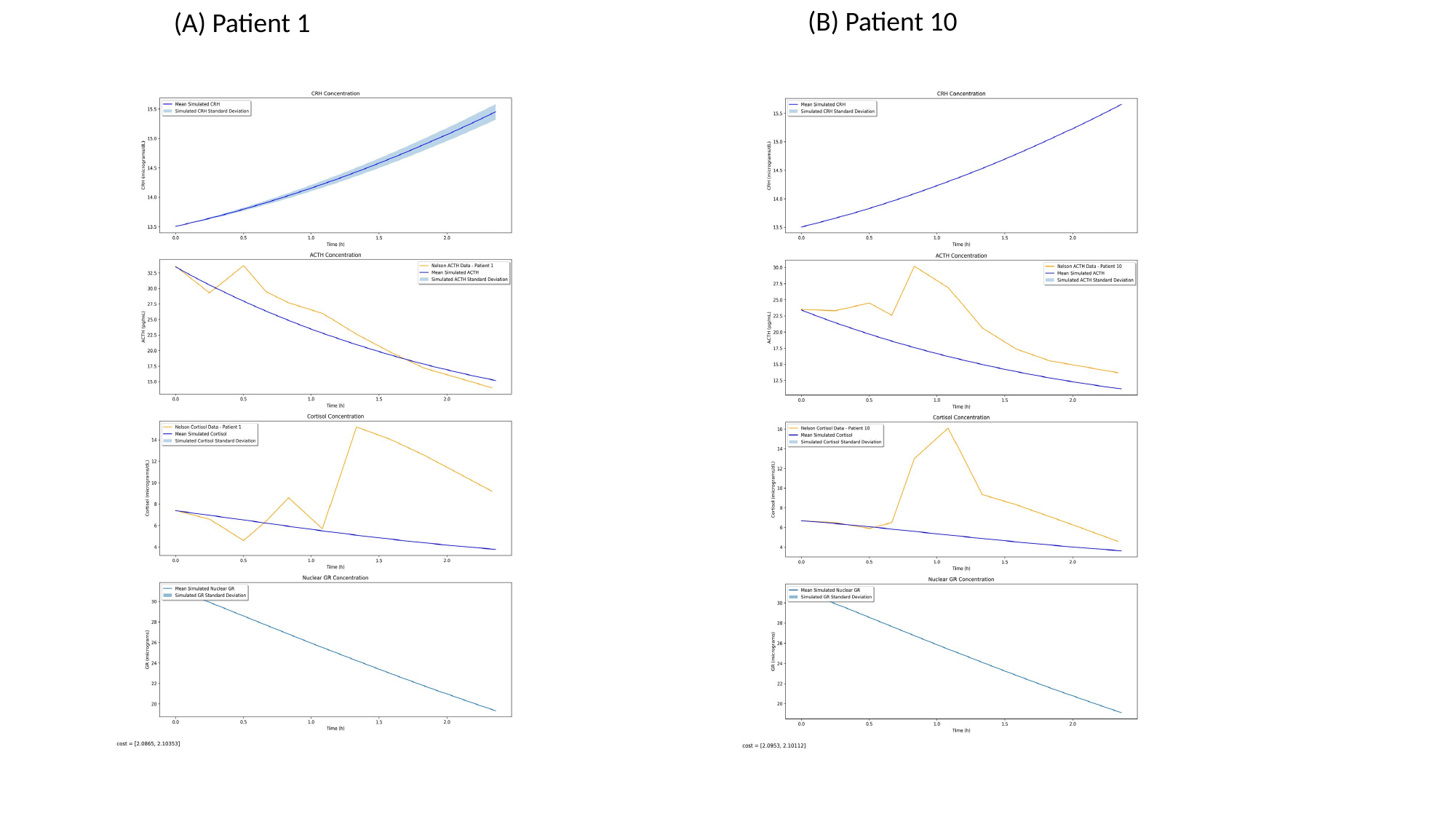

(A) Patient 1
(B) Patient 10

## Slide 27
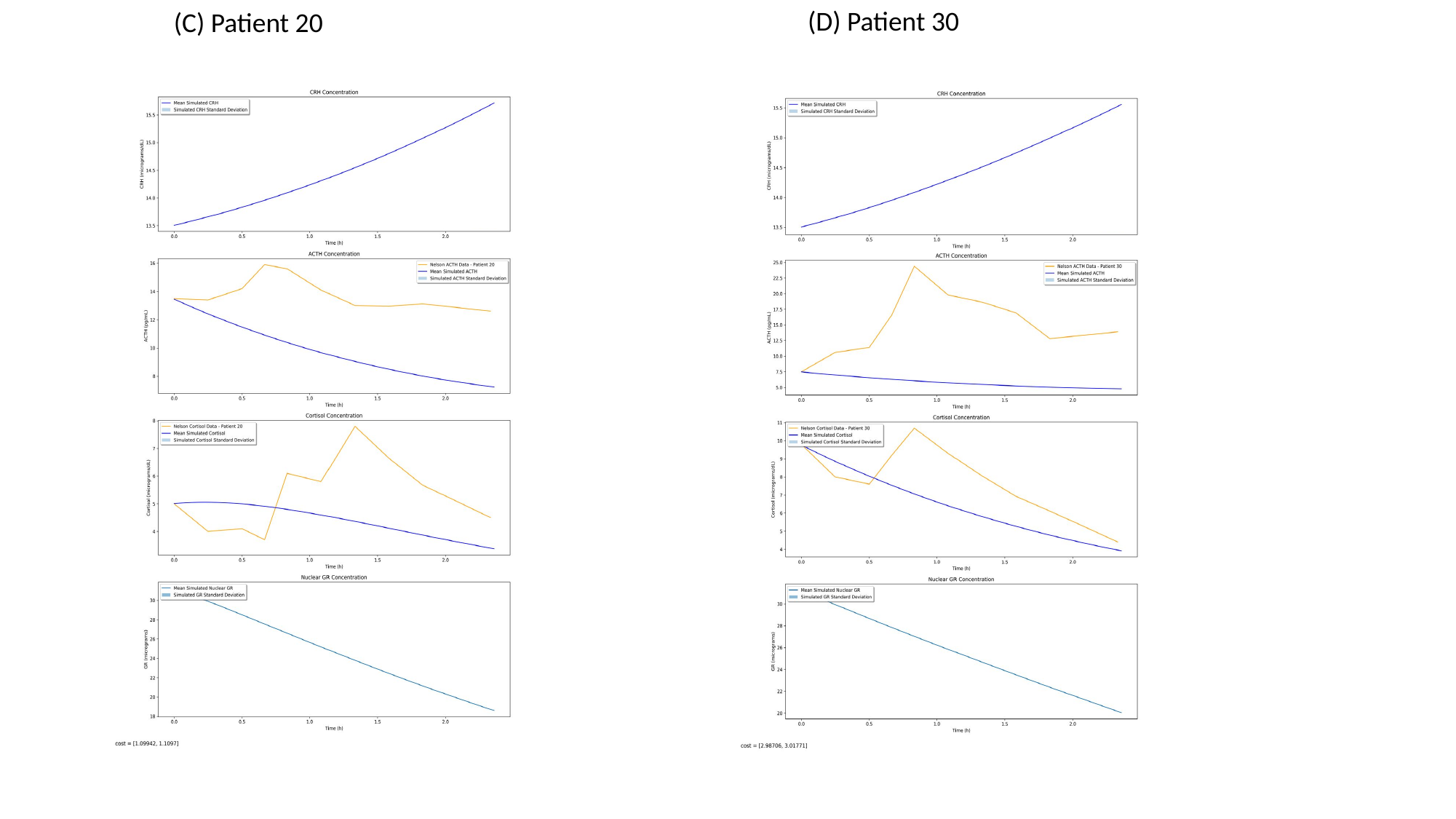

(C) Patient 20
(D) Patient 30

## Slide 28
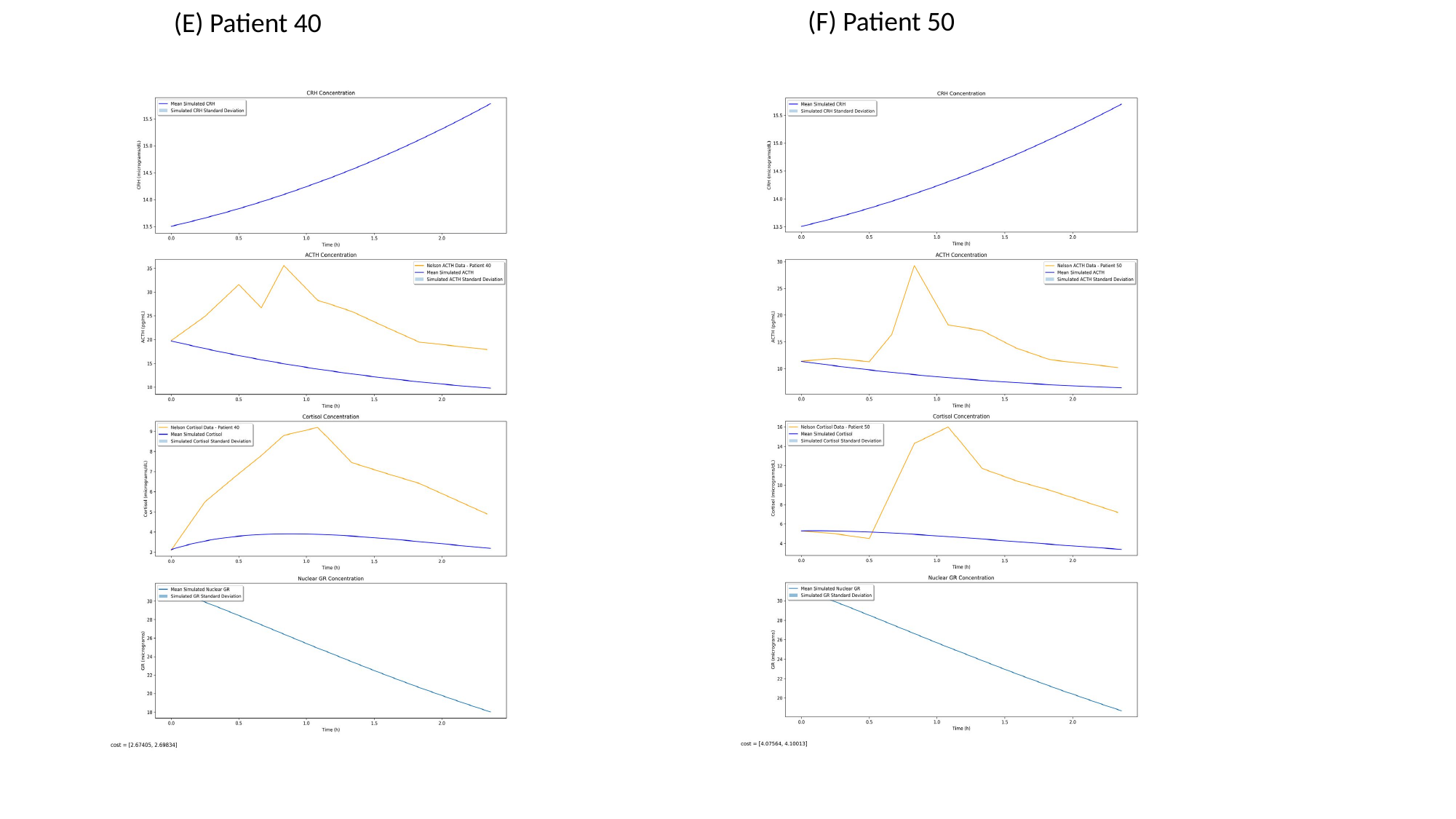

(E) Patient 40
(F) Patient 50

## Slide 29
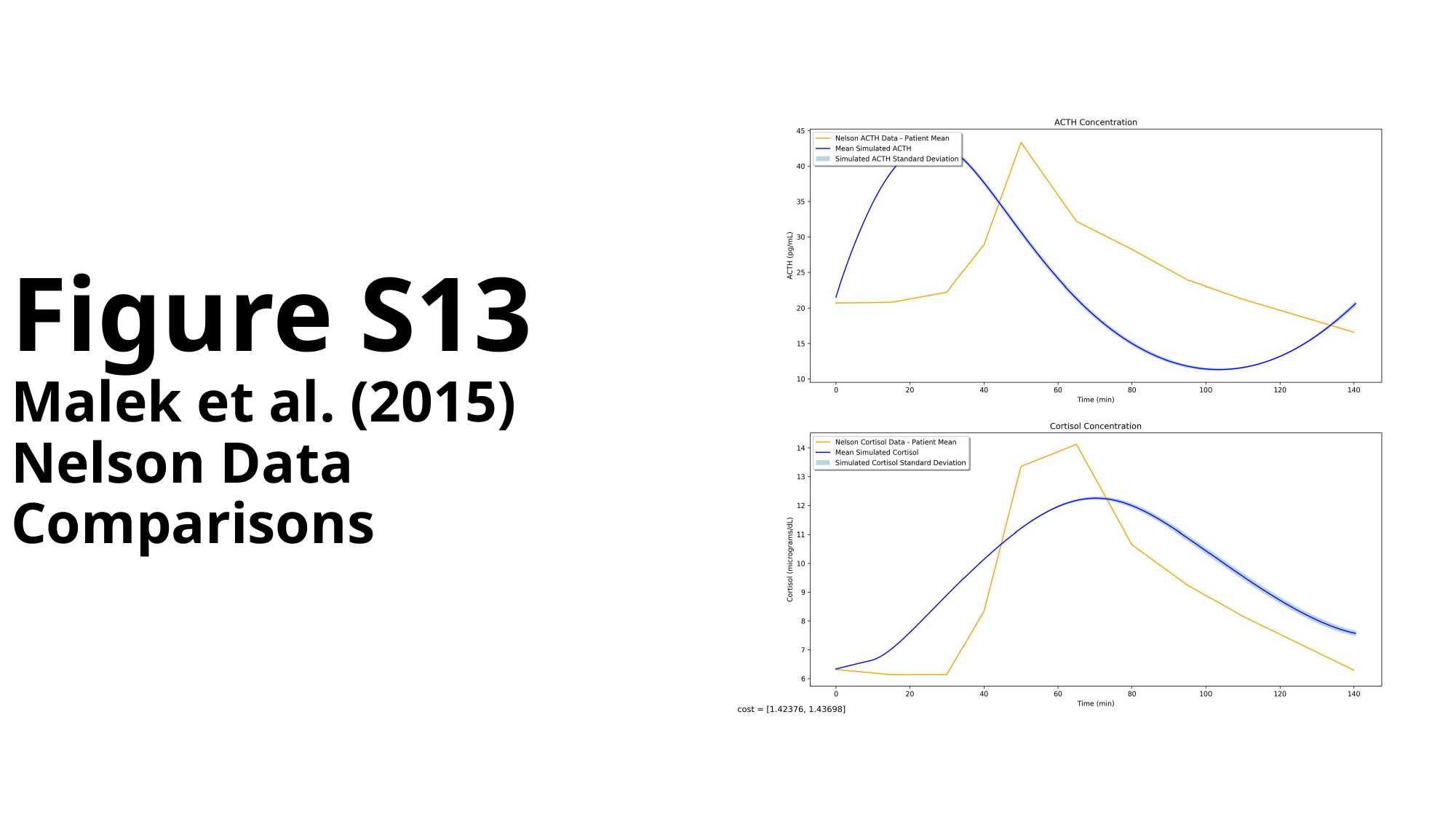

# Figure S13 Malek et al. (2015) Nelson Data Comparisons

## Slide 30
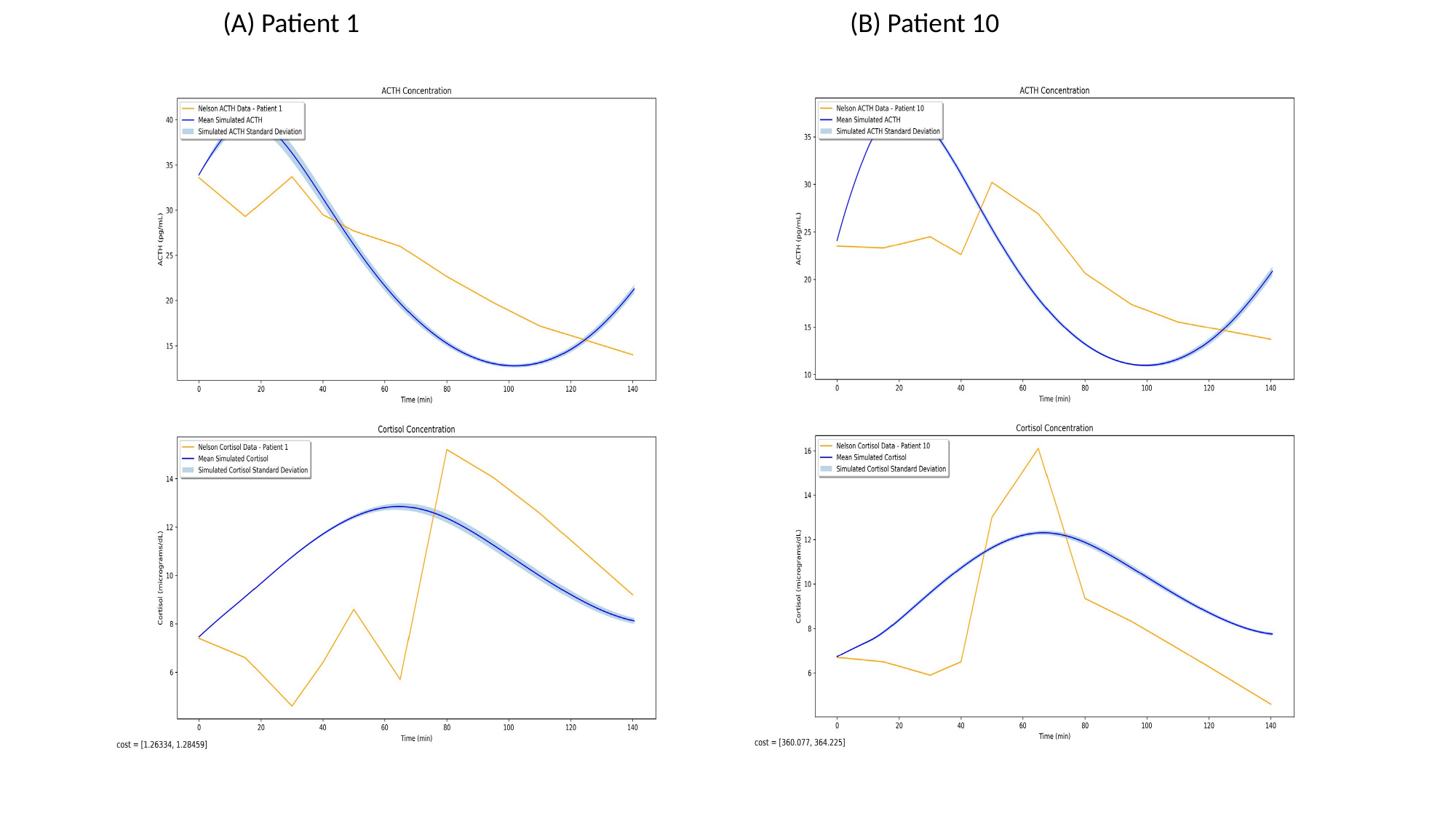

(A) Patient 1
(B) Patient 10

## Slide 31
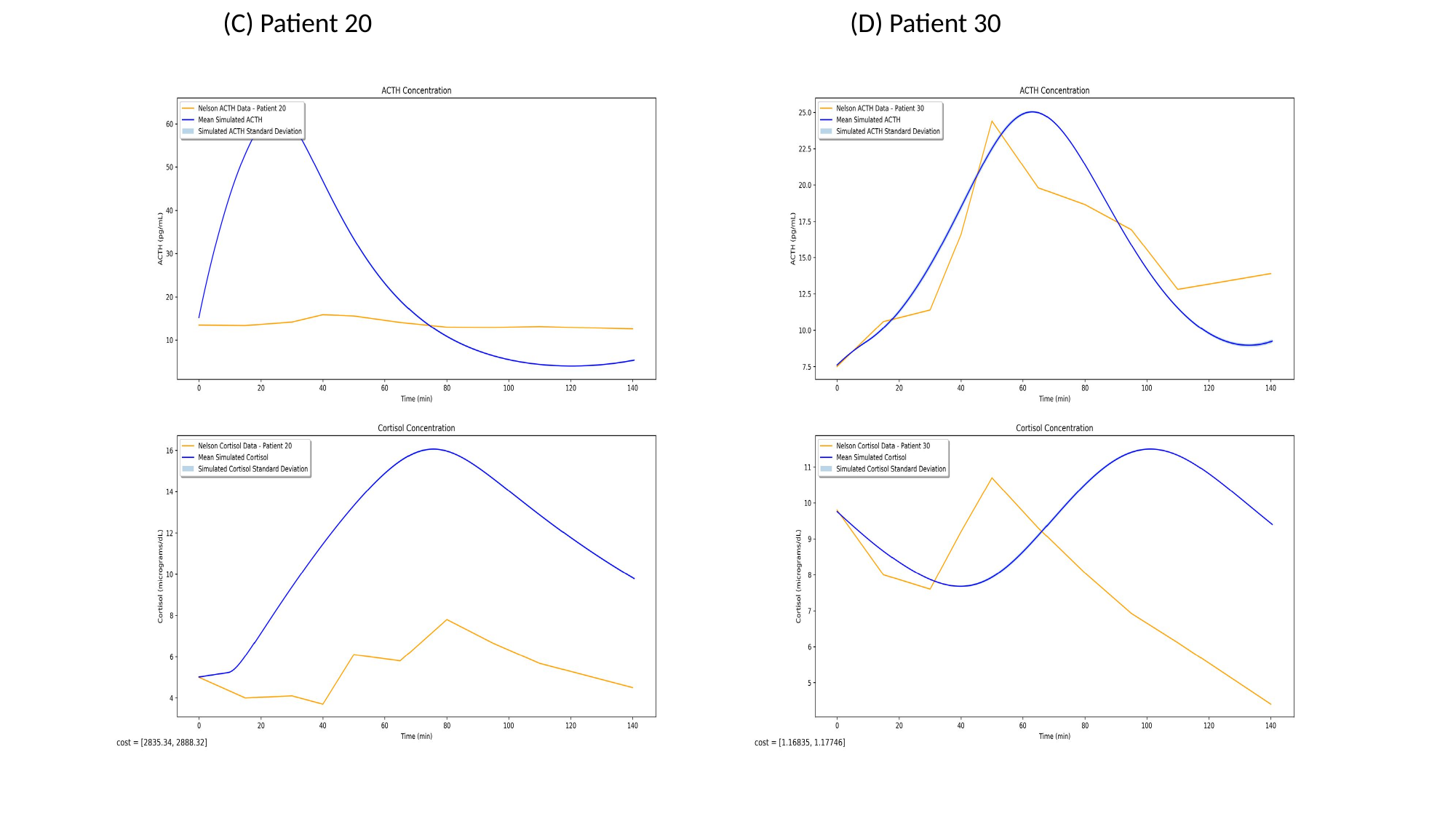

(C) Patient 20
(D) Patient 30

## Slide 32
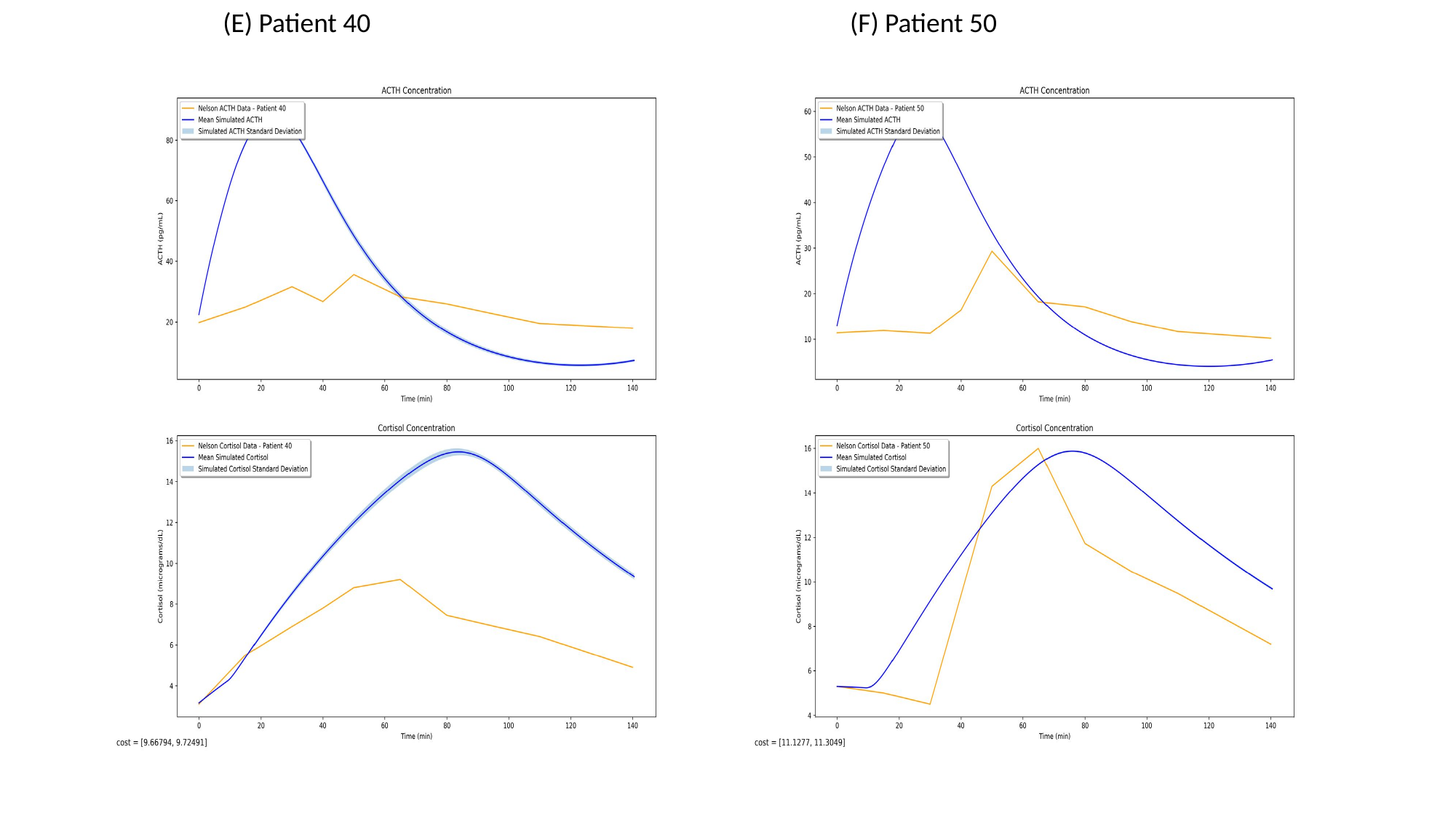

(E) Patient 40
(F) Patient 50

## Slide 33
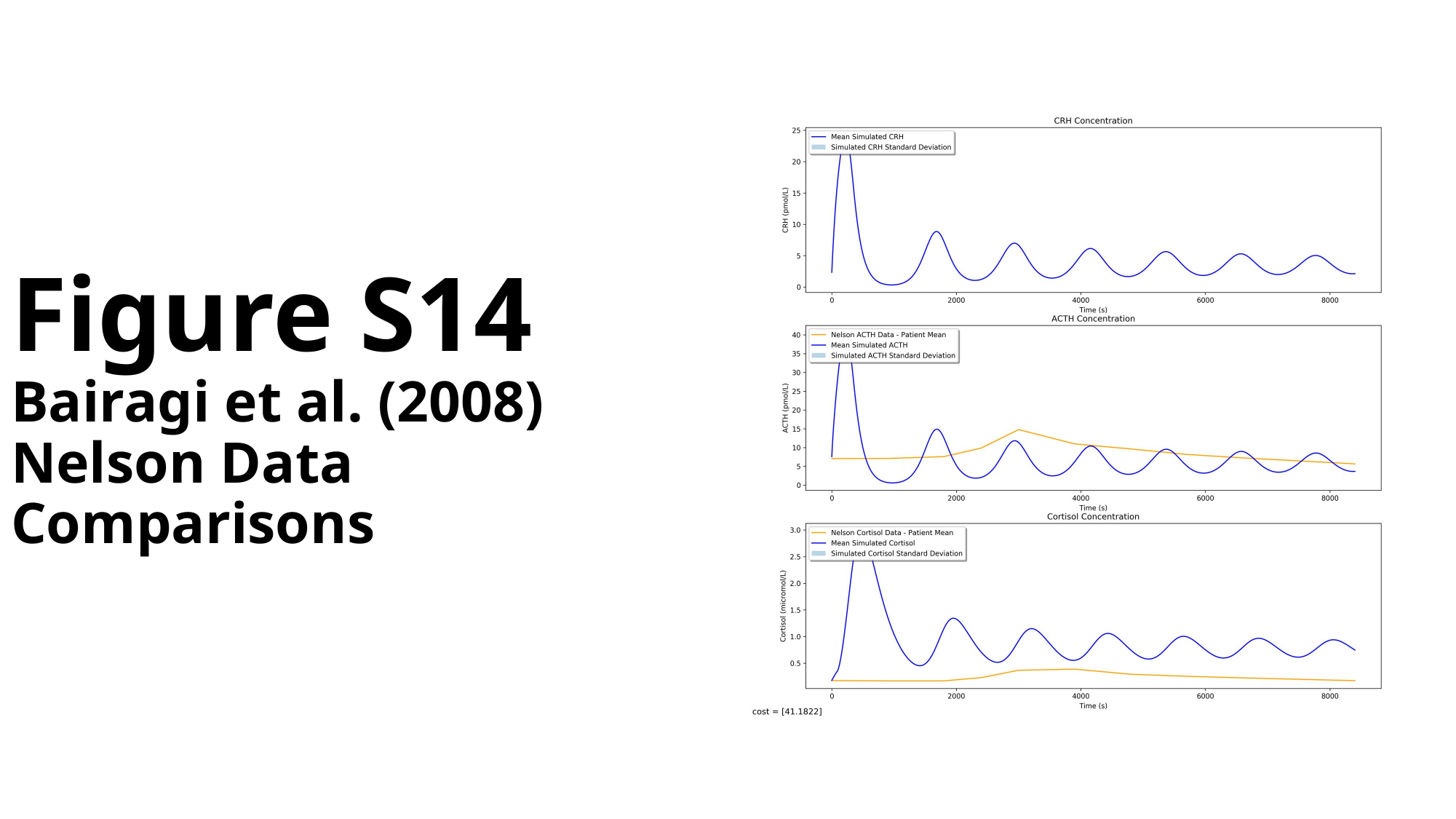

# Figure S14 Bairagi et al. (2008) Nelson Data Comparisons

## Slide 34
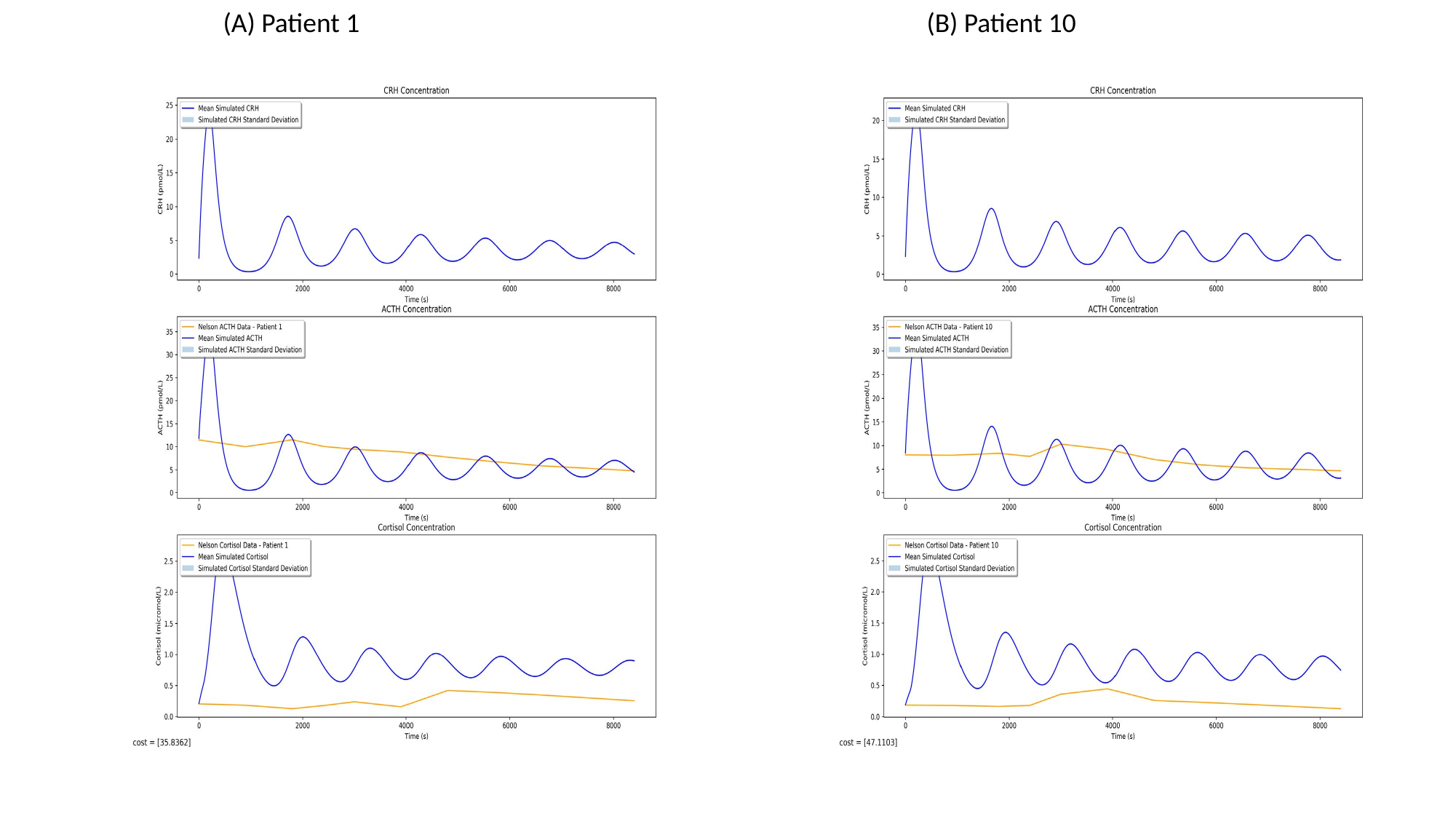

(A) Patient 1
(B) Patient 10

## Slide 35
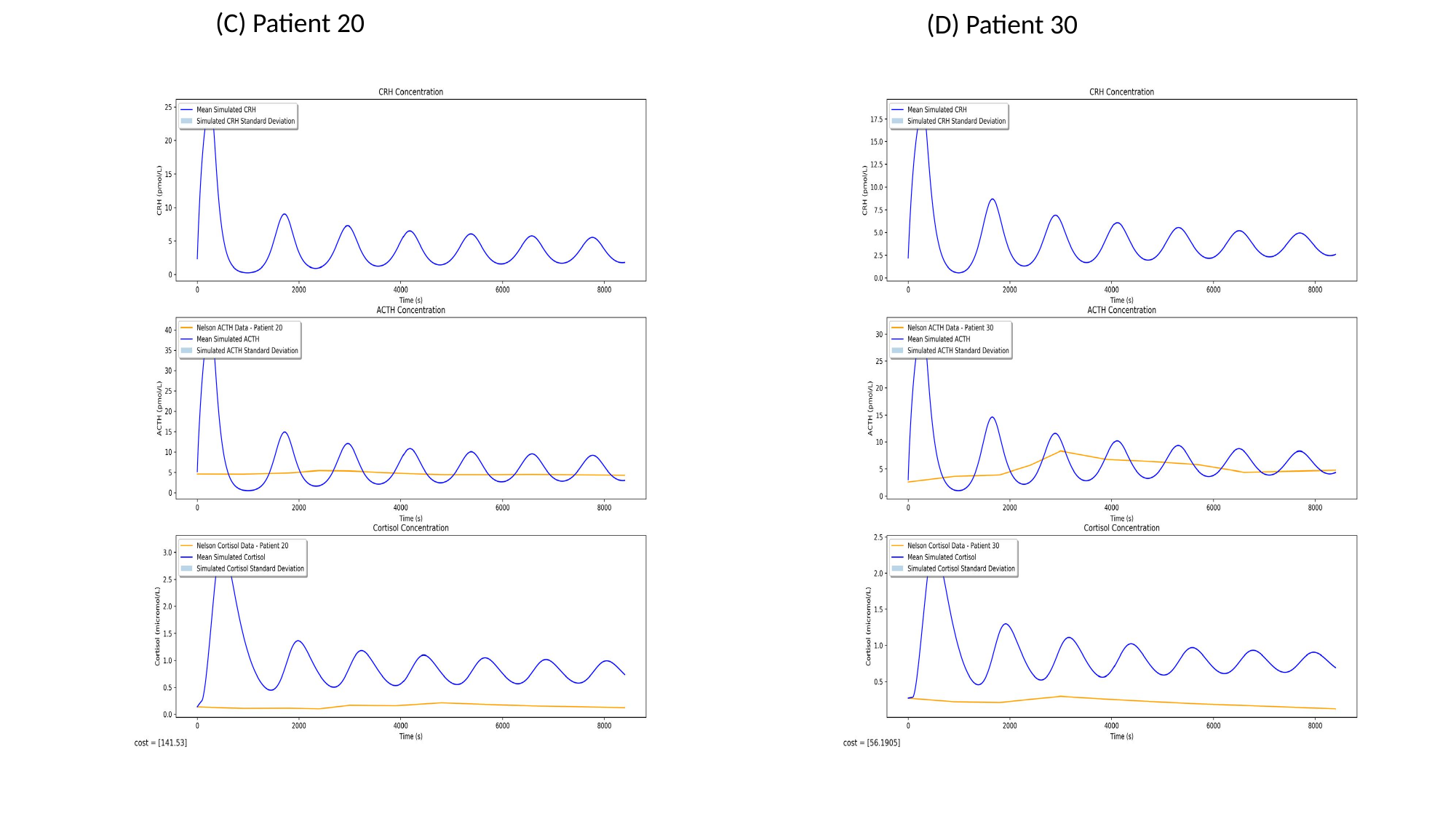

(C) Patient 20
(D) Patient 30

## Slide 36
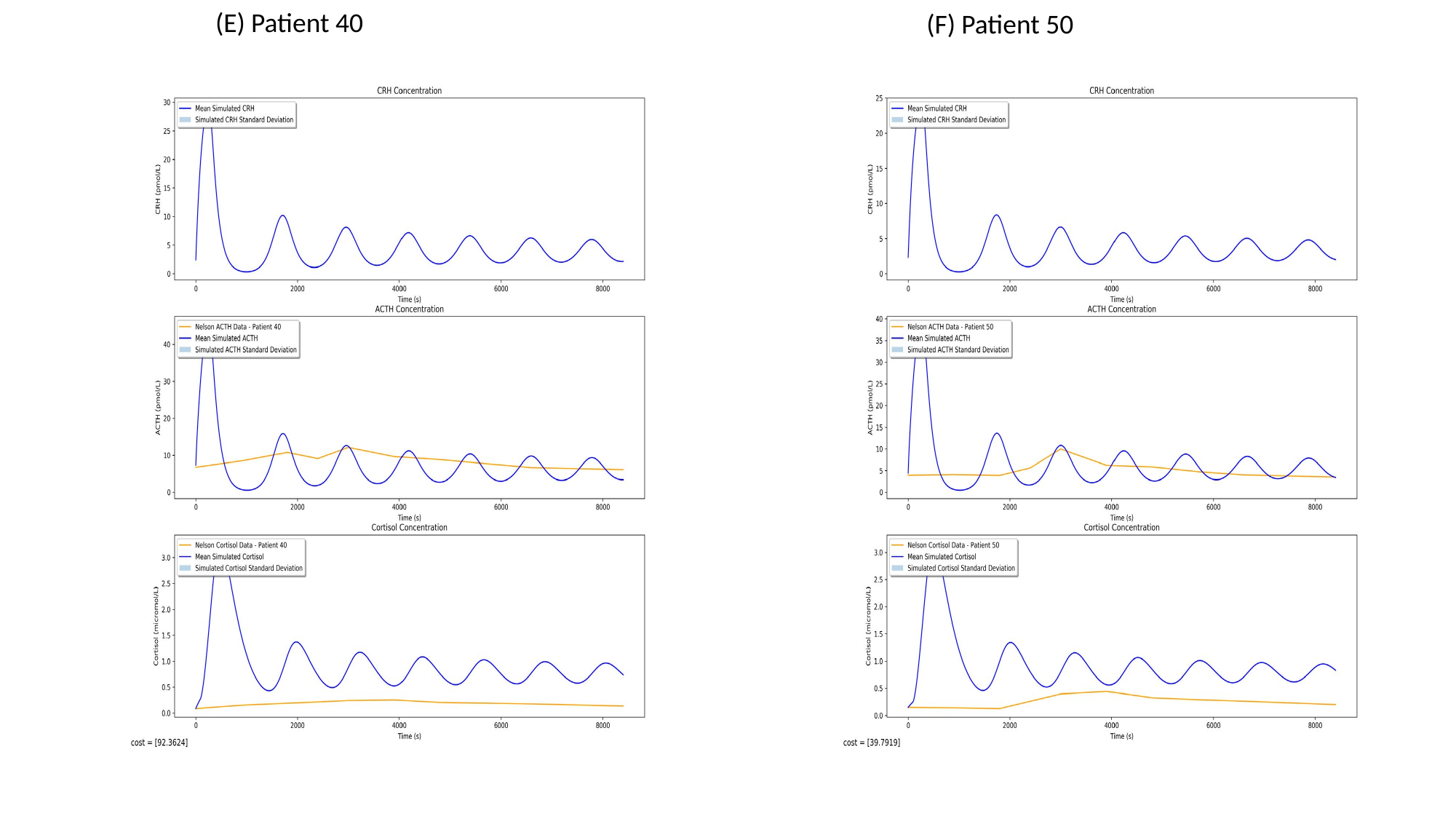

(E) Patient 40
(F) Patient 50
