# Supplementary material for: VeVaPy, a Python Platform for Efficient Verification and Validation of Systems Biology Models with Demonstrations Using Hypothalamic-Pituitary-Adrenal Axis Models
Source: Entropy (Basel). 2022 Nov 29;24(12):1747. doi: 10.3390/e24121747 (PMC9777964; doi:10.3390/e24121747)
Supplement: Supplementary file 1 [file entropy-24-01747-s001.zip › Supplementary Files/Supplementary-TablesS1-S3.pdf]

Supplementary Tables

Table S1. Revised DSM-IV criteria for melancholic subtype [1]

At least four of eight symptoms required for diagnosis	Non-reactive mood
	Severe anhedonia
	Distinct quality of mood
	Psychomotor disturbance
	Excessive guilt
	Early morning awakening
	Loss of appetite
	Mood worsening in the morning

Table S2. Revised DSM-IV for atypical subtype [1]

At least three of five symptoms required for diagnosis	Reactive mood
	Hypersomnia
	Hyperphagia or weight gain
	Leaden paralysis
	Rejection sensitivity

Table S3. Exclusion Criteria [1]

Any significant major medical illness
Diagnosis of a bipolar disorder
Current presence of significant suicidal ideation or a specific plan to harm themselves as indicated by a score of 3 or higher on the IDS question regarding suicidal ideation at any point in the study, or who have made a suicide attempt in the past month
Current diagnosis of social phobia due to public speaking fear
History of psychosis
History of substance dependence in the past year or meets criteria for a substance abuse disorder in the past three months
Currently pregnant or breast-feeding

Mental retardation
Subjects unable to give written informed consent
Any psychotropic medication in the past 1 week or antidepressant medication, including herbal or natural substances purported to have antidepressant properties (e.g. St. John's Wort, SAM-E, etc.) within 2 weeks (4 weeks for fluoxetine)
ECT within the last 6 months
Currently taking any medication that would interfere with HPA axis function (e.g. corticosteroids, ketoconazole, oral contraceptives, or exogenous estrogen for replacement therapy)
Current diagnosis of PTSD
Subjects with certain lifestyle habits (e.g. working night shift) that may affect the functioning of the HPA axis

1. Nelson, E.B., et al., *Hypothalamic-Pituitary-Adrenal Axis Dysregulation and Phenomenology of Major Depression*. 2008, University of Cincinnati.
