# Supplementary material for: VeVaPy, a Python Platform for Efficient Verification and Validation of Systems Biology Models with Demonstrations Using Hypothalamic-Pituitary-Adrenal Axis Models
Source: Entropy (Basel). 2022 Nov 29;24(12):1747. doi: 10.3390/e24121747 (PMC9777964; doi:10.3390/e24121747)
Supplement: Supplementary file 1 [file entropy-24-01747-s001.zip › Supplementary Files/TableS4-Andersen-2013-model-report.pdf]

Table S4. PBK Model Reporting Template Completed for Model by Andersen et al. (2013)

**PBK Model Reporting Template Sections**
A. **Name of Model**<br>*Mathematical Modeling of the Hypothalamic-Pituitary-Adrenal Gland (HPA) Axis, Including Hippocampal Mechanisms*
**B. Model author and contact details**<br>a. Morten Andersen—Technical University of Denmark, DTU Compute, Matematiktorvet 303B, 2800 Kongens Lyngby, Denmark; Roskilde Univesity, Building 27.1, NSM, IMFUFA, 4000 Roskilde, Denmark<br>b. Frank Vinther— Technical University of Denmark, DTU Compute, Matematiktorvet 303B, 2800 Kongens Lyngby, Denmark; Roskilde Univesity, Building 27.1, NSM, IMFUFA, 4000 Roskilde, Denmark<br>c. Johnny T. Ottesen (johnny@ruc.dk, tel: +45 4674 3020)— Roskilde Univesity, Building
**C. Summary of model characterization, development, validation and regulatory applicability**<br>Model scope is the interactions between CRH, ACTH and cortisol in the HPA axis, including the positive feedback by cortisol on CRH production via glucocorticoid receptors (GRs) in the hippocampus and its purpose is to simulate the ultradian oscillations observed in real-world ACTH and cortisol concentration data from human subjects. This model was developed as an extension of a model by the same authors in 2010, titled, "The minimal model of the hypothalamic-pituitary-adrenal axis."
**D. Model characterization**<br>a. **Scope and Purpose:** The scope is CRH, ACTH and cortisol concentrations including negative feedback by cortisol on CRH and ACTH (GRs in hypothalamus and pituitary, respectively) and positive feedback by cortisol on CRH (GRs in hippocampus). The model can be used for simulating these concentrations in the human HPA axis at baseline. The purpose was to simulate ultradian oscillations of CRH and ACTH (oscillations with period of ~1 hour) , although the model fails at this purpose. The model is mostly useful as an example of how hippocampal GR dynamics can be implemented in an ordinary differential equation (ODE) model of the HPA axis.<br>b. **Model Conceptualization:** The model consists of ODEs for CRH, ACTH and cortisol. These ODEs each consist of a production term and a degradation term. The CRH equation includes influence from hippocampal GRs and hypothalamic GRs (represented as terms in the form [CORT]/([CORT] + c) for different parameters c). The ACTH equation includes influence from pituitary GRs and from the current concentration of CRH. Finally, the cortisol equation includes production with influence by current ACTH concentration.<br>c. **Model Parameterization:** All parameters from the dimensional and non-dimensional forms of the model can be found in Tables 5.1 and 5.2 in the model paper, respectively. As the authors explain, the parameters are determined through, "an empirical guess … made through a study of the literature." We have used parameter

optimization to determine the most accurate parameters within +- 10% of the authors' published parameters.

d. **Computer Implementation:** The authors do not make clear the implementation they have used. We have used Python and our custom library called HPAmodeling that includes modules for ODE system solution and parameter optimization among other things. The parameter optimization was performed using the scipy.optimize.differential_evolution algorithm.

e. **Model Performance:** When attempting to simulate ultradian oscillations using the authors' published parameters, the performance of the model is unacceptable—because it does not succeed in producing ultradian oscillations. The model performs poorly when simulating patients undergoing Trier Social Stress Tests (TSSTs)—not surprisingly, as TSST data is outside the model's designed scope. See the paper for details on the performance of the model when attempting to simulate the HPA axis of subjects undergoing TSSTs.

f. **Model Documentation:** For documentation regarding the model, see the paper by Andersen et al. (2013), our paper or the model code (included in the Supplementary Materials along with this information).

E. Identification of uncertainties (report for each item in D.)

a. **Scope and Purpose:** N/A

b. **Model Conceptualization:** The model should likely include an ODE for GRs to compute the negative/positive feedback by cortisol. It would also potentially help if there was a function for creating varying stress inputs at different times during the simulation.

c. **Model Parameterization:** Because the parameters were determined by a best guess, rather than direct measurement, this is a large source of uncertainty. When we use parameter optimization, there is a degree of uncertainty in how wide the bounds on each parameter should be.

d. **Computer Implementation:** N/A

e. **Model Performance:** N/A

f. **Model Documentation:** N/A

F. Model implementation details (software used, availability of code)

The authors offer no insight into how they implemented the model during the research described in the paper. We programmed the model in Python using a custom library called HPAmodeling that contains modules for solving ODE and DDE systems and performing parameter optimization, among other modules. The model code and the HPAmodeling library are available at https://github.com/cparker-uc/VeVaPy.

G. Peer engagement (report extent of review by peers during development)

The authors offer no insight into the amount of peer review the model underwent during its creation.

H. Parameter tables (report all relevant inputs to the model for any simulations described)

See Table S4-1 below for dimensional system parameters and Table S4-2 below for dimensionless system parameters.

I. References and background information

See the paper referenced below for all background information and references used for creation of the model.

Andersen, M., F. Vinther, and J.T. Ottesen, *Mathematical modeling of the hypothalamic-pituitary-adrenal gland (HPA) axis, including hippocampal mechanisms.* Math Biosci, 2013. **246**(1): p. 122-38.

Table S4-1. Default parameter values for the dimensional system

**Parameter**	**Default Value**
k_0	0.859 pg/ml*min
k_1	0.127 1/min
k_2	0.00132 1/min
w_1	0.173 1/min
w_2	0.0348 1/min
w_3	0.009071 1/min
ρ	0.5
ψ	0.5
ξ	2
α	3
γ	3
c	3.06 ng/ml
c_3	1.42 ng/ml

Table S4-2. Default parameter values for the non-dimensional system

**Parameter**	**Default Value**
w_1	4.79
w_2	0.964
w_3	0.251
c_3	0.464
ψ	0.5

ρ	0.5
ξ	2
γ	3
α	3
