# Supplementary material for: VeVaPy, a Python Platform for Efficient Verification and Validation of Systems Biology Models with Demonstrations Using Hypothalamic-Pituitary-Adrenal Axis Models
Source: Entropy (Basel). 2022 Nov 29;24(12):1747. doi: 10.3390/e24121747 (PMC9777964; doi:10.3390/e24121747)
Supplement: Supplementary file 1 [file entropy-24-01747-s001.zip › Supplementary Files/TableS5-Bairagi-2008-model-report.pdf]

Table S5. PBK Model Reporting Template Completed for Model by Bairagi et al. (2008)

**PBK Model Reporting Template Sections**
**A. Name of model**<br>*Variability in the secretion of corticotropin-releasing hormone, adrenocorticotropic hormone and cortisol and understandability of the hypothalamic-pituitary-adrenal axis dynamics—a mathematical study based on clinical evidence*
**B. Model author and contact details**<br>**a.** N. Bairagi (nbairagi@math.jdvu.ac.in)—Centre for Mathematical Biology and Ecology, Department of Mathematics, Jadavpur University, Kolkata 700 032, India<br>**b.** Samrat Chatterjee (Samrat.ct@rediffmail.com)—Agricultural and Ecological Research Unit, Indian Statistical Institute, 203, B.T. Road, Kolkata 700 108, India<br>**c.** J. Chattopadhyay (joydev@isical.ac.in, corresponding author)—Agricultural and Ecological Research Unit, Indian Statistical Institute, 203, B.T. Road, Kolkata 700 108, India
**C. Summary of model characterization, development, validation and regulatory applicability**<br>This is a DDE-based model that includes equations for CRH, ACTH and cortisol. The equations for CRH and ACTH depend on the value of cortisol delayed by some time τ_2, and the equation for cortisol depends on ACTH delayed by some time τ_1. The delays are intended to introduce ultradian oscillations. The equation for CRH also includes a function to model the suprachiasmatic nucleus (SCN) of the hypothalamus—the main circadian clock in humans. This introduces circadian oscillations, as well.
**D. Model characterization**<br>a. **Scope and Purpose:** The scope of the model is modeling CRH, ACTH and cortisol concentrations with delays included between production of ACTH and cortisol and their actions. The actions of cortisol include negative feedback on CRH and ACTH. The CRH equation includes a function intended to model the SCN and introduce circadian oscillations. The purpose of the model is to demonstrate how delays between ACTH/cortisol production and action serve to introduce ultradian oscillations—if the magnitude of the delays is large enough. The model is also intended to predict the dynamics of the system after adrenalectomy or hypophysectomy, or on infusion of hormones.<br>b. **Model Conceptualization:** The model consists of 3 delay differential equations, one each for CRH, ACTH and cortisol. Each equation includes a degradation term that is a parameter multiplied by the current concentration of the hormone for that equation. The equations for CRH and ACTH contain nearly identical terms that influence production rate based on a parameter (the base rate of production, absent any cortisol in the system) and the concentration of cortisol at time $t - \tau_2$—this is how negative feedback by cortisol is introduced to the system. The rate of ACTH production also depends on the current concentration of CRH multiplied by a parameter. Finally, the production of cortisol depends on the concentration of ACTH at time $t - \tau_1$ multiplied by a parameter.

c. **Model Parameterization:** Elimination rates of the hormones were determined from the half-lives of the hormones. The other parameters were taken from available literature sources—the authors cite Jelic et al. (2005) as their source for parameter values.
d. **Computer Implementation:** The authors used MATLAB (version 6.5) to simulate the model as shown in their paper. We used Python with our custom HPAmodeling library, instead, as the authors did not provide the code they used for their simulations. The HPAmodeling library contains modules for solving ODE and DDE systems and running parameter optimizations (with the scipy.optimize.differential_evolution algorithm), among others.
e. **Model Performance:** The model performed the job for which it was designed well, as it is able to produce circadian and ultradian oscillations, and the performance under conditions intended to simulate adrenalectomy and hypophysectomy or hormone infusion aligns with our expectations of how the concentrations of CRH, ACTH and cortisol should perform in those situations. The performance of the model was much less adequate when used to simulate Major Depressive Disorder patients undergoing Trier Social Stress Tests (TSST). See our paper for details of this performance.
f. **Model Documentation:** The paper by Bairagi et al. (2008), cited at the end of this template, contains all documentation provided by the authors. For documentation regarding the use of the model to simulate TSST data, see our paper and the full code provided in the Supplementary Materials—the code is well-commented and these comments fully explain how the code works.

E. Identification of uncertainties (report for each item in D.)

a. **Scope and Purpose:** N/A
b. **Model Conceptualization:** The uncertainties in the model design come from the form in which cortisol feedback is introduced to the equations for CRH and ACTH. This feedback is simply introduced by dividing the production rate parameter by K + $[CORT]_{\tau 2}$ for a parameter K. This is less physiologically relevant than introducing an equation for glucocorticoid receptors and using this to produce the negative feedback by cortisol.
c. **Model Parameterization:** Uncertainty in the authors' published parameters comes mostly from the delays used for ACTH and cortisol. While the authors have tested multiple different delays for each and showing how this affects the behavior of the model, it is difficult to say exactly what these values should be. All of the delays used by the authors are longer than what is suggested in other papers where delays are tested. In our use of the model, we used parameter optimization to determine the most accurate parameter sets within certain bounds on the parameters. We based these bounds on the parameter values provided by the model authors +- 10%. It could be the case that this is too wide or too narrow a range for any or all of the parameters.
d. **Computer Implementation:** N/A
e. **Model Performance:** N/A
f. **Model Documentation:** N/A

**F. Model implementation details (software used, availability of code)**<br>The authors state that they used MATLAB version 6.5 to run simulations of the model with varying delays τ_1 and τ_2. They also ran simulations where the parameter governing cortisol production was set to 0 to simulate adrenalectomy, simulations where they added a second production term to the cortisol equation representing an infusion of synthetic cortisol, and a simulation where the parameter governing ACTH production was set to 0 to simulate hypophysectomy. We programmed the model in Python using a custom library called HPAmodeling that contains modules for solving ODE and DDE systems and performing parameter optimization, among other modules. The model code and the HPAmodeling library are available at https://github.com/cparker-uc/VeVaPy.
**G. Peer engagement (report extent of review by peers during development**<br>The authors offer no insight into the amount of peer review the model underwent during its creation.
**H. Parameter tables (report all relevant inputs to the model for any simulations described)**<br>See Table S5-1 below for parameter values and Table S5-2 below for delay values and their outcomes.
**I. References and background information**<br>See the paper referenced below for all background information and references used for creation of the model.<br><br>Bairagi, N., S. Chatterjee, and J. Chattopadhyay, *Variability in the secretion of corticotropin-releasing hormone, adrenocorticotropic hormone and cortisol and understandability of the hypothalamic-pituitary-adrenal axis dynamics--a mathematical study based on clinical evidence.* Math Med Biol, 2008. **25**(1): p. 37-63.<br><br>Jelic, S., Cupic, Z. & Kolar-Anic, L. (2005) *Mathematical modeling of the hypothalamic-pituitary-adrenal system activity*. Math. Biosci., **197**, p. 173–187.

Table S5-1. Variables and parameters used in the model

**Variable/Parameter**	**Definition**	**Default Value**
R	Concentration of CRH	Variable
A	Concentration of ACTH	Variable
C	Concentration of cortisol	Variable
b_1	Degradation rate constant of CRH	0.023
b_2	Degradation rate constant of ACTH	0.04
b_3	Degradation rate constant of cortisol	0.0083

g_1	Creation rate constant of ACTH	0.032
g_2	Creation rate constant of cortisol	0.0013
V	Positive constant	3
K	Positive constant	0.048
M	Hill coefficient	3
α_1	Positive constant	0.015
α_2	Positive constant	0.026

Table S5-2. Different combinations of delay and its outcomes

τ_1 (min)	τ_2 (min)	$\tau = \tau_1 + \tau_2$ (min)	**Theorem Satisfied**	**Stability**	**Figure number**
70	20	90	3.2	Limit cycle	4a
50	40	90	3.2	Limit cycle	4b
20	70	90	3.2	Limit cycle	4c
10	40	50	3.2	Asymptotically stable	5
20	30	50	3.2	Asymptotically stable	Not shown
40	10	50	3.2	Asymptotically stable	Not shown
