# Supplementary material for: VeVaPy, a Python Platform for Efficient Verification and Validation of Systems Biology Models with Demonstrations Using Hypothalamic-Pituitary-Adrenal Axis Models
Source: Entropy (Basel). 2022 Nov 29;24(12):1747. doi: 10.3390/e24121747 (PMC9777964; doi:10.3390/e24121747)
Supplement: Supplementary file 1 [file entropy-24-01747-s001.zip › Supplementary Files/TableS6-Bangsgaard-2018-model-report.pdf]

Table S6. PBK Model Reporting Template Completed for Model by Bangsgaard & Ottesen (2017)

**PBK Model Reporting Template Sections**
**A. Name of model**<br>*Patient specific modeling of the HPA axis related to clinical diagnosis of depression*
**B. Model author and contact details**<br>**a.** Elisabeth O. Bangsgaard—Department of Applied Mathematics and Computer Science, Technical University of Denmark, 2800 Kgs. Lyngby, Denmark<br>**b.** Johnny T. Ottesen (johnny@ruc.dk, corresponding author)—Department of Science and Environment, Roskilde University, Building 27.1, 4000 Roskilde, Denmark
**C. Summary of model characterization, development, validation and regulatory applicability**<br>The model is ODE-based with equations for CRH, ACTH and cortisol. The model has negative feedback by cortisol on CRH and ACTH, a circadian function representing the input of the suprachiasmatic nucleus (SCN) of the hypothalamus (the main circadian clock in humans) and an auto-up-regulating factor in CRH. The purpose of the model is to estimate parameters for individual patients in order to look for differences between depressed and control patients.
**D. Model characterization**<br>a. **Scope and Purpose:** The scope of the model is the concentrations of CRH, ACTH and cortisol in humans—including negative feedback by cortisol on CRH and ACTH, auto-up-regulation of CRH and circadian input on CRH production by the SCN. This can be used to simulate any human HPA axis at baseline, and it will produce circadian and ultradian oscillations. The purpose of the model is to estimate parameters for individual patients to find differences between patient populations (depressed and control, in the authors' work). While the model does well at this purpose in the authors' paper, we have been unable to exactly match their figures and replicate their work.<br>b. **Model Conceptualization:** The model consists of ODEs for CRH, ACTH and cortisol. Each equation is made up of a production term and a degradation term—all of the degradation terms are similar, with a parameter for each species multiplied by the current concentration of that species (for instance, $-w_1$*[CRH] for the CRH equation). The production term for CRH is a parameter plus three terms multiplied together. These are the circadian input function, the term for negative feedback by cortisol and the auto-up-regulation term (which is in the form [CRH]/(μ + [CRH])). The production term in the equation for ACTH is in the form a_3*[CRH]/(1 + a_4*[CORT]) so this accounts for the positive effect of CRH concentration and the negative effect of cortisol concentration. The equation for cortisol has production in the form a_5*$[ACTH]^2$, so it depends on the current concentration of ACTH squared. The circadian input function is dependent on the current time, and varies between ε and 1 for some parameter ε.<br>c. **Model Parameterization:** The parameters used were estimated for each individual patient data set, and the authors minimized a weighted sum of squares function as their cost function to determine the parameters. However, the authors do not provide any insight into how they determined bounds for each parameter to use in

the optimization nor which optimization algorithm was used. We also performed parameter optimization, using the authors' published parameter ranges where offered, and +- 10% of the average values of the authors' published parameters where necessary.

d. **Computer Implementation:** The authors do not provide any information about how they ran simulations with the model. We used Python and our custom library HPAmodeling (which contains modules for solving ODE and DDE systems and for parameter optimization, among others) to run simulations with the model. The parameter optimization was performed using the scipy.optimize.differential_evolution algorithm.

e. **Model Performance:** Based on the results published with the model, the authors were able to obtain good performance at matching basal concentrations of ACTH and cortisol for various individual patients (both depressed and control). However, we were unable to replicate the authors' results exactly, with model performance marginally worse when attempting to match the same data. When using the model to match data from Major Depressive Disorder and healthy control subjects undergoing Trier Social Stress Tests (TSST), the model performed poorly (much more poorly than when matching basal data)—this is to be expected as the TSST simulations fall outside the scope of the model's design.

f. **Model Documentation:** For documentation regarding the authors' use of the model, see the original paper by Bangsgaard & Ottesen (2018). For documentation regarding our use of the model, see the comments in the model code and our paper.

E. Identification of uncertainties (report for each item in D.)

a. **Scope and Purpose:** N/A

b. **Model Conceptualization:** The uncertainties in the model conceptualization arise due to the method in which negative feedback by cortisol arises—it would likely improve the model to include glucocorticoid receptors (GRs) and have these mediate the negative feedback loops. Also, the circadian input function is perhaps unnecessary to create circadian oscillations if the model includes GRs and/or delays between production of ACTH/cortisol and their action.

c. **Model Parameterization:** Due to the lack of information regarding how the authors arrived at the bounds used when performing their parameter optimization, we cannot be sure how physiologically reasonable these parameter values are. When we performed our parameter optimization, also, there is uncertainty in our bounds as a result of the uncertainty in the authors' published parameters and the fact that we cannot be sure that +- 10% is the appropriate range for all parameters.

d. **Computer Implementation:** N/A

e. **Model Performance:** N/A

f. **Model Documentation:** N/A

F. Model implementation details (software used, availability of code)

The authors offer no insight into how they implemented the model during the research described in the paper. We programmed the model in Python using a custom library called HPAmodeling that contains modules for solving ODE and DDE systems and performing

	parameter optimization, among other modules. The model code and the HPAmodeling library are available at https://github.com/cparker-uc/VeVaPy.
**G.**	**Peer engagement (report extent of review by peers during development)**<br>The authors offer no insight into the amount of peer review the model underwent during its creation.
**H.**	**Parameter tables (report all relevant inputs to the model for any simulations described)**<br>See Table S6-1 below.
**I.**	**References and background information**<br>See the paper referenced below for all background information and references used for creation of the model.<br><br>Bangsgaard, E.O. and J.T. Ottesen, *Patient specific modeling of the HPA axis related to clinical diagnosis of depression.* Math Biosci, 2017. **287**: p. 24-35.

Table S6-1. The values of the estimated parameters and the remaining fixed parameters for control and hypercortisolemic depressed subjects

	**Control**	**Hyper**	**p-value**	**H_0**	**Unit**
a_0	$4.71 \times 10^{-2} \pm 4.89 \times 10^{-2}$	$1.31 \times 10^{-1} \pm 8.25 \times 10^{-2}$	4.47×10^{-2}	1	pg/(mL*min)
a_1	$6.84 \times 10^{12} \pm 6.89 \times 10^{9}$	$1.29 \times 10^{13} \pm 1.55 \times 10^{12}$	4.81×10^{-5}	1	pg/(mL*min)
a_2	1.78×10^{9}	1.78×10^{9}	-	-	$(dL/\mu g)^2$
μ	583	583	-	-	pg/mL
a_3	2.28×10^{4}	2.28×10^{4}	-	-	min^{-1}
a_4	1.77×10^{5}	1.77×10^{5}	-	-	dL/μg
a_5	$3.81 \times 10^{-4} \pm 2.39 \times 10^{-4}$	$3.03 \times 10^{-4} \pm 1.47 \times 10^{-4}$	4.82×10^{-1}	0	$(\mu g/dL)/(min*(pg/mL)^2)$
ω_1	$4.49 \times 10^{-2} \pm 1.22 \times 10^{-2}$	$4.57 \times 10^{-2} \pm 1.25 \times 10^{-2}$	9.04×10^{-1}	0	min^{-1}
ω_2	$2.25 \times 10^{-2} \pm 1.47 \times 10^{-2}$	$1.46 \times 10^{-2} \pm 4.82 \times 10^{-3}$	2.16×10^{-1}	0	min^{-1}
ω_3	$2.01 \times 10^{-2} \pm 1.11 \times 10^{-2}$	$2.10 \times 10^{-2} \pm 5.89 \times 10^{-3}$	8.67×10^{-1}	0	min^{-1}
δ	$8.61 \times 10^{2} \pm 6.91 \times 10^{2}$	$2.01 \times 10^{1} \pm 1.79 \times 10^{1}$	1.81×10^{-2}	1	min
α	300	300	-	-	min
k	5	5	-	-	-
β	950	950	-	-	min
l	6	6	-	-	-
ε	0.01	0.01	-	-	-
N_c	0.5217	0.5217	-	-	-
