# Supplementary material for: VeVaPy, a Python Platform for Efficient Verification and Validation of Systems Biology Models with Demonstrations Using Hypothalamic-Pituitary-Adrenal Axis Models
Source: Entropy (Basel). 2022 Nov 29;24(12):1747. doi: 10.3390/e24121747 (PMC9777964; doi:10.3390/e24121747)
Supplement: Supplementary file 1 [file entropy-24-01747-s001.zip › Supplementary Files/TableS7-Malek-2015-model-report.pdf]

Table S7. PBK Model Reporting Template Completed for Model by Malek et al. (2015)

PBK Model Reporting Template Sections

A. Name of model

Dynamics of the HPA axis and inflammatory cytokines: Insights from mathematical modeling

B. Model author and contact details

a. Hamed Malek—Biocomputing Laboratory, Computer and Information Technology Engineering Department, Amirkabir University of Technology, Tehran, Iran

b. Mohammad Mehdi Ebadzadeh (ebadzadeh@aut.ac.ir, corresponding author)—Biocomputing Laboratory, Computer and Information Technology Engineering Department, Amirkabir University of Technology, Tehran, Iran

c. Reza Safabakhsh— Biocomputing Laboratory, Computer and Information Technology Engineering Department, Amirkabir University of Technology, Tehran, Iran

d. Alireza Razavi—Department of Immunology, School of Public Health, Tehran University of Medical Sciences, Tehran, Iran

Jalal Zaringhalam—Neurophysiology Research Center, Department of Physiology, Shahid Beheshti University of Medical Sciences, Tehran, Iran

C. Summary of model characterization, development, validation and regulatory applicability

This delay differential equation (DDE) model consists of 5 equations, allowing simulation of the following species: ACTH, cortisol, endotoxin, TNF-α and IL-6. The model can be used as a model of the HPA axis only by setting the initial values of the inflammatory cytokines and endotoxin to 0. The purpose of the model is to illustrate how the HPA axis influences inflammation—the authors state that it "captures the main qualitative features of cytokine and hormone dynamics when a toxic challenge is introduced."

D. Model characterization

a. **Scope and Purpose:** The scope is ACTH and cortisol concentrations, including delays in ACTH/cortisol action after release, negative feedback by cortisol on ACTH and inflammatory cytokine interactions after introduction of endotoxins to the system. The model can generate ultradian oscillations and the model also includes a circadian input function in the equation for ACTH to introduce circadian oscillations. The purpose of the model is to examine "the role of key inflammatory cytokines and cortisol in transition from acute to persistent inflammation through stability analysis."

b. **Model Conceptualization:** The model consists of 5 delay differential equations, each consisting of 0-2 production terms and a degradation term. The degradation terms are all similar and consist of a parameter for each species times the current concentration of that species (for instance, the degradation term of ACTH is -e_A*[ACTH]). The production terms for ACTH and cortisol include positive feedback from the cytokines. The equation for ACTH contains negative feedback from cortisol in the form c/(c + [CORT]) for a parameter c. The equation for cortisol contains positive influence from ACTH concentration in the form α*[ACTH]^m/(a + [ACTH]^m). The endotoxin equation has no production terms, instead having 2 degradation terms with the second one containing negative feedback from TNF-α and IL-6. The TNF-α has positive influence from itself and endotoxin and negative influence from IL-6 and

cortisol. Finally, the IL-6 equation has positive influence from TNF-α and negative influence from cortisol. The circadian input function in the ACTH equation is in the form $c_1 + c_2*\sin(c_3*t) - c_4*\cos(c_5*t)$.

c. **Model Parameterization:** The majority of the parameters were determined by estimation or guessing, according to the authors. For parameter estimation, the authors used an algorithm called real-coded estimation of distribution algorithm (RCEDA). A total of 10 of 32 parameters have sources reported for the values used in the authors' simulations. We have used parameter optimization to determine the most accurate parameter sets for simulating ACTH and cortisol concentrations of patients undergoing Trier Social Stress Tests (TSSTs). The bounds we used were the authors' published parameter values $\pm$ 10%.

d. **Computer Implementation:** The authors provide no information about how they ran simulations of the model. We used Python with our custom library HPAmodeling (containing modules for solving ODEs and DDEs and for parameter optimization with the scipy.optimize.differential_evolution algorithm, among other modules) to perform simulations to match TSST data.

e. **Model Performance:** The model performs well in the authors' publication, and we were able to recreate this performance in our own simulations with their published parameters. The model performance was significantly worse when simulating patient concentrations while undergoing TSSTs—unsurprisingly, as these simulations are outside the model's designed scope.

f. **Model Documentation:** For documentation of the authors' simulations, see the paper by Malek et al. (2015). For documentation of our simulations against TSST data, see our paper or the comments included in our model code.

E. Identification of uncertainties (report for each item in D.)

a. **Scope and Purpose:** N/A

b. **Model Conceptualization:** The uncertainties in the model conceptualization arise from the lack of an equation for CRH, thereby making the circadian input function potentially less physiologically valid. Furthermore, the model would be made more accurate with the inclusion of glucocorticoid receptors to mediate the negative feedback by cortisol on ACTH (and CRH, if it is included).

c. **Model Parameterization:** The authors' estimation of parameters is a source of uncertainty, as they do not make clear the bounds used during the procedure. Further, the 5 parameters for which the values are listed as guesses introduce a large amount of uncertainty. Our parameter optimization during our simulations of TSST data is also somewhat uncertain, since we have used the authors' published parameter values $\pm$10% for bounds, and this could be too small or large for any of the parameters.

d. **Computer Implementation:** N/A

e. **Model Performance:** N/A

f. **Model Documentation:** N/A

F. Model implementation details (software used, availability of code)

	The authors offer no insight into how they implemented the model during the research described in the paper. We programmed the model in Python using a custom library called HPAmodeling that contains modules for solving ODE and DDE systems and performing parameter optimization, among other modules. The model code and the HPAmodeling library are available at https://github.com/cparker-uc/VeVaPy.
**G.**	**Peer engagement (report extent of review by peers during development)**<br>The authors offer no insight into the amount of peer review the model underwent during its creation.
**H.**	**Parameter tables (report all relevant inputs to the model for any simulations described)**<br>See Table S7-1 below.
**I.**	**References and background information**<br>See the paper referenced below for all background information and references used for creation of the model.<br><br>Malek, H., et al., *Dynamics of the HPA axis and inflammatory cytokines: Insights from mathematical modeling.* Comput Biol Med, 2015. **67**: p. 1-12.<br><br>References from the paper by Malek et al. (2015) used in the parameter table:<br><br>[1] M. Andersen, F. Vinther, J.T. Ottesen, *Mathematical modeling of the hypothalamicpituitary-adrenal gland (HPA) axis, including hippocampal mechanisms*, Math. Biosci. 246(1 (November)) (2013) 122–138. ISSN: 1879-3134.http://dx.doi.org/10.1016/j.mbs.2013.08.010.<br><br>[6] B.J. Carroll et al., *Pathophysiology of hypercortisolism in depression*, Acta Psych. Scand. 433 (Supplementum) (2007), 90–103. ISSN: 0065-1591. http:// dx.doi.org/10.1111/j.1600-0447.2007.00967.x.<br><br>[9] J. Day et al., *A reduced mathematical model of the acute inflammatory response II. Capturing scenarios of repeated endotoxin administration*, J. Theor. Biol. 242 (1 (September)) (2006), 237–256. ISSN: 0022-5193. 〈http://dx. doi.org/10.1016/j.jtbi.2006.02.015〉.<br><br>[32] J.C. Oliver et al., *Cytokine kinetics in an in vitro whole blood model following an endotoxin challenge*, Lymphokine Cytokine Res. 12 (2 (April)) (1993) 115-120. ISSN: 1056-5477<br><br>[46] C. Weigert et al., *Upregulation of IL-6 mRNA by IL-6 in skeletal muscle cells: role of IL-6 mRNA stabilization and Ca2 þ -dependent mechanisms*, Am. J. Physiol. – Cell Physiol. 293 (3 (September)) (2007) C1139–C1147 (visited on 11/ 23/2013). http://dx.doi.org/10.1152/ajpcell.0

Table 1. Parameter Values

Param	Value	Unit	Source
e_P	0.05	min^{-1}	[9]
e_T	0.038	min^{-1}	[32]
e_S	0.02	min^{-1}	[46]
e_A	0.04	min^{-1}	[46]
e_C	0.01	min^{-1}	[6]
d_1	0.026	min^{-1}	est.
d_2	0.068	$(\mu g/dl)(ng/cl)(min)^{-1}$	est.
d_3	0.063	$(ng/cl)^2(min)^{-1}$	est.
d_4	2.37	$(\mu g/dl)(ng/cl)(min)^{-1}$	est.
d_5	9.39	$(pg/ml)(min)^{-1}$	est.
d_6	0.35	$(\mu g/dl)(min)^{-1}$	est.
K	0.0504	$(ng/cl)(min)^{-1}$	est.
τ_1	10	min	Guess
τ_2	10	min	Guess
m_1	4	-	Guess
m_2	4	-	Guess
c	6.11	μg/dl	[6]
a	21	pg/ml	[6]
h	7.66	$(pg/ml)(min)^{-1}$	[1]
α	0.28	$(\mu g/dl)(min)^{-1}$	Guess
x_1	3.25	ng/cl	est.
x_2	0.86	ng/cl	est.
x_3	0.016	IU/kg	est.
x_4	6.11	μg/dl	[6]
x_5	1.39	$(ng/cl)^2$	est.
x_6	1.57	ng/cl	est.
x_7	6.11	μg/dl	[6]

x_8	1.72	ng/cl	est.
x_9	0.87	ng/cl	est.
x_{10}	0.94	ng/cl	est.
x_{11}	1.87	ng/cl	est.
x_{12}	1.97	ng/cl	est.
