# Supplementary material for: VeVaPy, a Python Platform for Efficient Verification and Validation of Systems Biology Models with Demonstrations Using Hypothalamic-Pituitary-Adrenal Axis Models
Source: Entropy (Basel). 2022 Nov 29;24(12):1747. doi: 10.3390/e24121747 (PMC9777964; doi:10.3390/e24121747)
Supplement: Supplementary file 1 [file entropy-24-01747-s001.zip › Supplementary Files/TableS8-Somvanshi-2020-model-report.pdf]

Table S8. PBK Model Reporting Template Completed for Model by Somvanshi et al. (2020)

PBK Model Reporting Template Sections

A. Name of model

Role of enhanced glucocorticoid receptor sensitivity in inflammation in PTSD: Insights from a computational model for circadian-neuroendocrine-immune interactions

B. Model author and contact details

- **a.** Pramod R. Somvanshi—Harvard John Paulson School of Engineering and Applied Sciences, Harvard University, Cambridge, MA
- **b.** Synthia H. Mellon—Department of Obstetrics, Gynecology & Reproductive Sciences, University of California, San Fransisco, CA
- **c.** Rachel Yehuda—Department of Psychiatry, James J. Peters VA Medical Center, Bronx, NY; Department of Psychiatry, Icahn School of Medicine at Mount Sinai, NY
- **d.** Janine D. Flory— Department of Psychiatry, James J. Peters VA Medical Center, Bronx, NY; Department of Psychiatry, Icahn School of Medicine at Mount Sinai, NY
- **e.** Linda Bierer— Department of Psychiatry, James J. Peters VA Medical Center, Bronx, NY; Department of Psychiatry, Icahn School of Medicine at Mount Sinai, NY
- **f.** Iouri Makotkine— Department of Psychiatry, James J. Peters VA Medical Center, Bronx, NY; Department of Psychiatry, Icahn School of Medicine at Mount Sinai, NY
- **g.** Charles Marmar—Department of Psychiatry, New York Langone Medical School, New York, NY
- **h.** Marti Jett—Integrative Systems Biology, US Army Medical Research and Materiel Command, USACEHR, Fort Detrick, Frederick, MD
- **i.** Francis J. Doyle III (frank_doyle@seas.harvard.edu, corresponding author)—Harvard John Paulson School of Engineering and Applied Sciences, Harvard University, Cambridge, MA

C. Summary of model characterization, development, validation and regulatory applicability

This model is an ODE model with 17 equations describing the concentrations of CRH, ACTH, cortisol, glucocorticoid receptors (GRs), and also inflammatory cytokines such as IL-6, IL-10 and TNF-α. The model is capable of demonstrating the relationship between the HPA axis and inflammation.

D. Model characterization

a. **Scope and Purpose:** The scope of the model is much wider than the other HPA axis models we have tested, simply because it includes more equations covering more compounds in the HPA axis and inflammatory system. The model is capable of demonstrating the interactions between CRH, ACTH and cortisol—including the binding of cortisol to GRs to cause negative feedback on the production of CRH and ACTH—and it also demonstrates the interactions between lipopolysaccharides, phagocytes, TGF, TNF-α, IL-10 and IL-6 (as well as the GR mediated action of cortisol on them). Also included in the model is the ability to introduce dexamethasone to the system and simulate the effects of a dexamethasone suppression test (DST) on the HPA axis and the immune response. The purpose of the model is to determine

differences between PTSD subjects and healthy controls in terms of their inflammatory response.

b. **Model Conceptualization:** The model consists of 17 ordinary differential equations. These equations describe the rates of change of the following: CRH, ACTH, StAR protein, cortisol, dexamethasone (compartment 1), dexamethasone (plasma), cortisol (delayed action), glucocorticoid receptor mRNA, glucocorticoid receptor protein, glucocorticoid receptors (cytosolic), glucocorticoid receptors (nuclear), lipopolysaccharides, phagocytes, TGF, TNF-α, IL-10, and IL-6. Generally, these equations consist of a production term and a degradation term, similar in nature to other HPA axis models. For more information about the structure of the model equations, see the paper by Somvanshi et al. (2020), as the model is much too large to describe fully here.

c. **Model Parameterization:** The model has a total of 67 parameters. The values used by the authors came from three different sources: 15 parameters are “estimated in current study. Recalibrated from (Sriram et al., 2012) and (Bangsgaard et al., 2017)”, 10 parameter values come from Rao et al. (2016), and 42 parameter values are from Bangsgaard et al. (2017). We used a parameter optimization algorithm to determine the most accurate parameter sets for simulating the HPA axes of patients undergoing Trier Social Stress Tests (TSSTs). For parameter bounds, we used the authors’ published parameters $\pm$ 10%.

d. **Computer Implementation:** On the first author’s Github, a model substantially similar to this model is published although it was difficult to find and is not linked to by the paper. The program for running simulations with a similar model is written in MATLAB. We have run simulations using Python.

e. Model Performance: The model performs well when simulating the interactions between the HPA axis and the immune response at baseline or during/after dexamethasone administration. However, the performance of the model is reduced when simulating patients undergoing TSSTs. See our paper for more details on this performance.

f. Model Documentation: For documentation regarding the model, see the paper by Somvanshi et al. (2020), our paper or the model code (included in the Supplementary Materials along with this information).

E. Identification of uncertainties (report for each item in D.)

a. Scope and Purpose: N/A

b. Model Conceptualization: The uncertainty in the model conceptualization arises from the extreme complexity of the model. With each added equation or parameter, the added uncertainty increases, so 17 equations and 67 parameters is a large amount of uncertainty. The model would likely benefit from an analysis to determine if the results could be reached with a simpler model.

c. Model Parameterization: The uncertainty in the model parameterization arises from the 15 parameters that are estimated based on the papers by Bangsgaard et al. (2017) and Sriram et al. (2012). The authors do not give any information about which parameters in these papers they are correlating with the parameters in this paper and how they are recalibrating them to fit this circumstance. Unfortunately, this

introduces a lot of uncertainty into the authors' published parameter values. We used the authors' parameter values when running our parameter optimizations, using ± 10% for bounds on each parameter. This compounds this uncertainty, as we are also unsure that the bounds should be exactly this size, it is simply a reasonable guess.

d. **Computer Implementation:** N/A
e. **Model Performance:** N/A
f. **Model Documentation:** N/A

F. Model implementation details (software used, availability of code)
The authors used MATLAB, and we were able to find a substantially similar model on the first author's GitHub. However, this was not mentioned in the paper and was difficult to find. We programmed the model in Python using a custom library called HPAmodeling that contains modules for solving ODE and DDE systems and performing parameter optimization, among other modules. The model code and the HPAmodeling library are available at https://github.com/cparker-uc/VeVaPy.

G. Peer engagement (report extent of review by peers during development)
The authors offer no insight into the amount of peer review the model underwent during its creation.

H. Parameter tables (report all relevant inputs to the model for any simulations described)
See Table S8-1 below.

I. References and background information
See the paper referenced below for all background information and references used for creation of the model.

Somvanshi, P.R., et al., *Role of enhanced glucocorticoid receptor sensitivity in inflammation in PTSD: insights from computational model for circadian-neuroendocrine-immune interactions.* Am J Physiol Endocrinol Metab, 2020. **319**(1): p. E48-E66.

Bangsgaard, E.O., Hjorth, P.G., Olufsen, M.S., Mehlsen, J., and Ottesen, J.T. (2017). Integrated Inflammatory Stress (ITIS) Model. Bull Math Biol 79, 1487-1509.

Rao, R., DuBois, D., Almon, R., Jusko, W.J., and Androulakis, I.P. (2016). Mathematical modeling of the circadian dynamics of the neuroendocrine- immune network in experimentally induced arthritis. Am J Physiol Endocrinol Metab 311, E310-E324.

Sriram, K., Rodriguez-Fernandez, M., and Doyle III, F.J. (2012). Modeling Cortisol Dynamics in the Neuro-endocrine Axis Distinguishes Normal, Depression, and Post-traumatic Stress Disorder (PTSD) in Humans. PLoS Comput Biol 8, e1002379.

Table S8-1. Parameter Values

Parameter	Value	Units	Ref.
N	1	-	Estimated in current study. Recalibrated from (Sriram et al., 2012) and (Bangsgaard et al., 2017)
Ki_{n1}	1.2	nM/ml	Estimated
K_{strs}	1	µg/dL/min	Estimated
n3	2	-	Estimated
Vs3	0.032	1/min	Estimated
Kp2	0.41	1/min	Estimated
Vs4	0.016	1/min	Estimated
Vs5	0.0266	1/min	Estimated
kp4	4.5×10^{-5}	1/min	Estimated
q5	40	pg/mL	Estimated
q6	12	pg/mL	Estimated
q7	2	pg/mL/min	Estimated
q8	40	pg/mL	Estimated
m	4	-	Estimated
Cirmax	4	-	Estimated
$ksynt_{rm}$	3.625	1/h	Rao et al., 2016
nkm_{Grn}	26	nM/mg protein	Rao et al., 2016
$kdeg_{rm}$	0.1124	1/h	Rao et al., 2016
$ksynt_{rp}$	1.2	1/h	Rao et al., 2016
vp_rp	0.0279	1/h	Rao et al., 2016
kon	0.00329	nM/h	Rao et al., 2016
deg_rp	0.0572	1/h	Rao et al., 2016
krt	0.63	1/h	Rao et al., 2016
kre	0.57	1/h	Rao et al., 2016

km	50	nM	Rao et al., 2016
kphg	4.9956×10^{7}	kg/h/pg	Bangsgaard et al., 2017
kptnf	12.94907	-	Bangsgaard et al., 2017
xnTNF	1693.951	pg/ml	Bangsgaard et al., 2017
xTGF	0.07212	pg/ml	Bangsgaard et al., 2017
xIL10	147.68	pg/ml	Bangsgaard et al., 2017
dpg	0.144	1/h	Bangsgaard et al., 2017
ktgf	1.56×10^{-9}	ml/pg*U*h	Bangsgaard et al., 2017
dtgf	0.03177	1/h	Bangsgaard et al., 2017
ktnf	25.5194	pg/ml*h	Bangsgaard et al., 2017
TNFn	5.5×10^{6}	U	Bangsgaard et al., 2017
xtntg	0.1589	pg/ml	Bangsgaard et al., 2017
ktnff	3.5514×10^{4}	pg/ml*h	Bangsgaard et al., 2017
dtnf	0.0307	ml/pg*h	Bangsgaard et al., 2017
sil	1187.2	pg/ml*h	Bangsgaard et al., 2017
xil	8.0506×10^{7}	U	Bangsgaard et al., 2017
dil	98.932	1/h	Bangsgaard et al., 2017
xild	791.27	pg/ml	Bangsgaard et al., 2017
kiltg	43875	pg/ml*h	Bangsgaard et al., 2017
xiltg	0.38	pg/ml	Bangsgaard et al., 2017
vtnf	1	nM/h	Bangsgaard et al., 2017
kp_{tnf}	10	pg/ml	Bangsgaard et al., 2017
xil6il6	1.987×10^{5}	pg/ml	Bangsgaard et al., 2017
xil6il10	1.1818	pg/ml	Bangsgaard et al., 2017
xil6tgf	4.23	pg/ml	Bangsgaard et al., 2017
dil6	0.43605	1/h	Bangsgaard et al., 2017
il6b	0.4	pg/ml*h	Bangsgaard et al., 2017

q1	0.5	ml/pg*h	Bangsgaard et al., 2017
q2	625	μg/dL	Bangsgaard et al., 2017
q3	2.8014	pg/ml*min	Bangsgaard et al., 2017
q4	112	pg/ml*min	Bangsgaard et al., 2017
kIL10	267480	pg/ml*h	Bangsgaard et al., 2017
kil10il6	1.1188	pg/ml*h	Bangsgaard et al., 2017
xIL10IL6	26851	pg/ml	Bangsgaard et al., 2017
kil6	$1.0x10^{3}$	pg/ml*h	Bangsgaard et al., 2017
xil6	$1.1x10^{5}$	pg/ml	Bangsgaard et al., 2017
kil6tnf	4.4651	pg/h*ml*U	Bangsgaard et al., 2017
xil6tnf	1211.3	pg/ml	Bangsgaard et al., 2017
lt	$1.0x10^{-6}$	U	Bangsgaard et al., 2017
kil6il6	122.92	$1/h*U^{-4}$	Bangsgaard et al., 2017
kmtnf	100	pg/ml	Bangsgaard et al., 2017
dlp	$1.35x10^{-7}$	1/h*U	Bangsgaard et al., 2017
nx	1	-	Bangsgaard et al., 2017
