# Supplementary material for: VeVaPy, a Python Platform for Efficient Verification and Validation of Systems Biology Models with Demonstrations Using Hypothalamic-Pituitary-Adrenal Axis Models
Source: Entropy (Basel). 2022 Nov 29;24(12):1747. doi: 10.3390/e24121747 (PMC9777964; doi:10.3390/e24121747)
Supplement: Supplementary file 1 [file entropy-24-01747-s001.zip › Supplementary Files/TableS9-Sriram-2012-model-report.pdf]

*Table S9. PBK Model Reporting Template for Model by Sriram et al. (2012)*

| PBK Model Reporting Template Sections |
|---|
| **A. Name of model**<br>*Modeling Cortisol Dynamics in the Neuro-endocrine Axis Distinguishes Normal, Depression, and Post-traumatic Stress Disorder (PTSD) in Humans* |
| **B. Model author and contact details**<br>**a.** K. Sriram—Institute of Collaborative Biotechnologies, University of California, Santa Barbara, California, United States of America; Department of Chemical Engineering, University of California, Santa Barbara, California, United States of America<br>**b.** Maria Rodriguez-Fernandez—Institute of Collaborative Biotechnologies, University of California, Santa Barbara, California, United States of America; Department of Chemical Engineering, University of California, Santa Barbara, California, United States of America<br>**c.** Francis J. Doyle III (doyle@engineering.ucsb.edu, corresponding author)—Institute of Collaborative Biotechnologies, University of California, Santa Barbara, California, United States of America; Department of Chemical Engineering, University of California, Santa Barbara, California, United States of America |
| **C. Summary of model characterization, development, validation and regulatory applicability**<br>Model scope encompasses the HPA axis including CRH, ACTH, cortisol and cortisol-bound glucocorticoid receptors (GRs). The inclusion of GRs allows for more accurate feedback by cortisol on CRH and ACTH. Using a bifurcation analysis of the model, the authors attempt to determine differences in parameters between depressed, PTSD and healthy control subjects. |
| **D. Model characterization**<br>a. **Scope and Purpose:** The scope is CRH, ACTH, cortisol and GR concentrations with GRs facilitating negative feedback by cortisol on CRH and ACTH (from hypothalamic and pituitary GRs, respectively). The model can produce circadian and (under ideal conditions) ultradian oscillations without needing a function to represent input from the suprachiasmatic nucleus (SCN) or delays between ACTH/cortisol production and action. The purpose of the model is to examine differences between depressed, PTSD and healthy control subjects in terms of the amount of stress input to the CRH equation necessary to produce large cortisol responses.<br>b. **Model Conceptualization:** The model consists of four ordinary differential equations, one each for CRH, ACTH, cortisol and GRs. The equations for CRH, ACTH and cortisol consist of one production term each and two degradation terms each. The production terms for CRH and ACTH are in the form of $k_1/(k_2 + [GR]^n)$ which introduces the negative feedback by cortisol (through the GRs). The degradation terms are one enzymatic, Michaelis-Menten form and one standard degradation term of the form $-k_d$*[concentration]. The equation for cortisol has the same form of degradation terms and the production term is in the form k*[ACTH]. The equation for GR consists of two production terms and a single degradation term (in the form $-k_d$*[GR]). One production term is a Michaelis-Menten term and the other is in the form k*[CORT]*([$G_{tot}$] – [GR]). $G_{tot}$ includes both bound and unbound glucocorticoid |

receptors, and GR is only the receptor complex with cortisol, so ($[G_{tot}]$ – [GR]) is total unbound glucocorticoid receptors. So GR production increases in terms of the concentration of cortisol and the concentration of unbound glucocorticoid receptors.

c. **Model Parameterization:** All model parameters except for two (n2 and $G_{tot}$) include references for values and bounds. The authors perform parameter optimization for all parameters and report the optimized values along with the literature values. We use the authors' published bounds for our own parameter optimization.

d. **Computer Implementation:** The authors use XPPAUT for bifurcation analysis of the model and MATLAB for the remaining figures. We use Python with our custom HPAmodeling library for simulations with the model.

**e.** **Model Performance:** The model performs very well when simulating the HPA axis at baseline. It can produce circadian and ultradian oscillations. The performance is also acceptable when we use it to simulate subjects undergoing Trier Social Stress Tests (TSSTs), although this is outside of its designed scope, although it has room for improvement.

**f.** **Model Documentation:** For documentation regarding the model, see the paper by Sriram et al. (2012), our paper or the model code (included in the Supplementary Materials along with this information).

**E. Identification of uncertainties (report for each item in D.)**

**a.** **Scope and Purpose:** N/A

**b.** **Model Conceptualization:** The lack of delays between production and action for ACTH and cortisol and the lack of SCN input to the system are introducing some uncertainty.

**c.** **Model Parameterization:** The two parameters with values that are "assumed" rather than coming from sources in the literature introduce significant uncertainty. The inclusion of reasonable bounds for each parameter from the literature allows for less uncertainty with parameter optimization, so there is only a small amount of uncertainty in the authors' optimized parameter values and our optimized parameter values.

**d.** **Computer Implementation:** N/A

**e.** **Model Performance:** N/A

**f.** **Model Documentation:** N/A

**F. Model implementation details (software used, availability of code)**

The authors used XPPAUT for bifurcation analysis and MATLAB for all other simulations with the model. We programmed the model in Python using a custom library called HPAmodeling that contains modules for solving ODE and DDE systems and performing parameter optimization, among other modules. The model code and the HPAmodeling library are available at https://github.com/cparker-uc/VeVaPy.

**G. Peer engagement (report extent of review by peers during development)**

The authors offer no insight into the amount of peer review the model underwent during its creation.

**H. Parameter tables (report all relevant inputs to the model for any simulations described)**

See Table S9-1 below.

## I. References and background information

See the paper referenced below for all background information and references used for creation of the model.

Sriram, K., M. Rodriguez-Fernandez, and F.J. Doyle, 3rd, *Modeling cortisol dynamics in the neuro-endocrine axis distinguishes normal, depression, and post-traumatic stress disorder (PTSD) in humans.* PLoS Comput Biol, 2012. **8**(2): p. e1002379.

*Table S9-1. Estimated kinetic parameters of normal, PTSD and depressed subjects used in the bifurcation analysis and numerical integration*

| Constants | Literature Values | Source and Ref | Lower Bound | Upper Bound | Optimized Values |
|---|---|---|---|---|---|
| $k_{stress}$ (Normal) | 0.76 µM $h^{-1}$ | vs [19] | 5 | 20 | 10.1 µg $dL^{-1}$ $h^{-1}$ |
| $k_{stress}$ (Depressed) | 0.76 µM $h^{-1}$ | vs [19] | 5 | 20 | 13.7 µg $dL^{-1}$ $h^{-1}$ |
| $k_{stress}$ (PTSD) | 0.76 µM $h^{-1}$ | vs [19] | 5 | 20 | 17.5 µg $dL^{-1}$ $h^{-1}$ |
| $k_i$ (Normal) | 1 µM | k1 [19] | 0.5 | 3 | 1.51 µg $dL^{-1}$ |
| $k_i$ (Depressed) | 1 µM | k1 [19] | 0.5 | 3 | 1.60 µg $dL^{-1}$ |
| $k_i$ (PTSD) | 1 µM | k1 [19] | 0.5 | 3 | 1.17 µg $dL^{-1}$ |
| $V_{S3}$ | 1.58 – 5 µM $h^{-1}$ | v1-v4 [19] | 3 | 4 | 3.25 µg $dL^{-1}$ $h^{-1}$ |
| $K_{m1}$ | 2 µM | k1-k4 [19] | 1 | 2 | 1.74 µg $dL^{-1}$ |
| $K_{P2}$ | 0.3 – 1.8 $h^{-1}$ | ks/k1 [19] | 7 | 11 | 8.30 $h^{-1}$ |
| $V_{S4}$ | 1.58 – 5 µM $h^{-1}$ | v1-v4 [19] | 0.5 | 1.5 | 0.907 µg $dL^{-1}$ $h^{-1}$ |
| $K_{m2}$ | 2 µM | k1-k4 [19] | 0.08 | 2 | 0.112 µg $dL^{-1}$ |
| $K_{P3}$ | 0.3 – 1.8 $h^{-1}$ | ks/k1 [19] | 0.5 | 1.2 | 0.945 $h^{-1}$ |
| $V_{S5}$ | 1.58 – 5 µM $h^{-1}$ | v1-v4 [19] | 0.001 | 0.008 | 0.00535 µg $dL^{-1}$ $h^{-1}$ |
| $K_{m3}$ | 2 µM | k1-k4 [19] | 0.03 | 0.08 | 0.0768 µg $dL^{-1}$ |
| $K_{d1}$ | 0.173 $min^{-1}$ | CRH degradation [28] | 0.002 | 0.005 | 0.00379 $h^{-1}$ |
| $K_{d2}$ | 0.035 $min^{-1}$ | ACTH degradation [28] | 0.001 | 0.01 | 0.00916 $h^{-1}$ |
| $K_{d3}$ | 0.009 $min^{-1}$ | CORT degradation [28] | 0.1 | 0.5 | 0.356 $h^{-1}$ |
| $n_1$ | 5 | n [41,42] | 4 | 6 | 5.43 |

| | | | | | |
|---|---|---|---|---|---|
| $n_2$ | 4 | Assumed | 4 | 6 | 5.10 |
| $K_b$ | - | [41,42] | 0.008 | 0.05 | 0.0202 $h^{-1}$ |
| $G_{tot}$ | Not known | Assumed | 2 | 5 | 3.28 μg |
| $V_{S2}$ | 0 – 1 (arb. units) | vs2 [41,42] | 0.01 | 0.07 | 0.0509 μg $dL^{-1}$ $h^{-1}$ |
| K1 | 1 (arb. units) | k1 [41,42] | 0.2 | 0.7 | 0.645 μg $dL^{-1}$ |
| $K_{d5}$ | 0.01 (arb. units) | kd5 [41,42] | 0.04 | 0.09 | 0.0854 $h^{-1}$ |
